# Resolving aromatic spectral congestion in whole-cell solid-state NMR: Signatures of condensed tannins and lignin in plant tissues

**DOI:** 10.1016/j.tcsw.2026.100182

**Published:** 2026-09-22

**Authors:** Priya Sahu, Peng Xiao, Debkumar Debnath, Jayasubba Reddy Yarava, Qinghui Cheng, Tuo Wang

**Affiliations:** a Department of Chemistry, Michigan State University, East Lansing, MI 48824, USA; b State key Laboratory of Non-food Biomass Energy Technology, Institute of Biological Science and Technology, Guangxi Academy of Sciences, Nanning 530007, China

**Keywords:** Cell wall, Plant, Solid-state NMR, Lignin, Tannin, Polysaccharides, Chemical shift

## Abstract

Solid-state NMR enables direct characterization of intact biological materials but is often hindered by spectral congestion arising from chemically similar components. A major challenge in plant tissues is distinguishing condensed tannins from lignin, two aromatic polymers that frequently coexist and generate overlapping ^13^C resonances. Here, we establish experimentally validated spectral signatures for differentiating condensed tannins from lignin in whole-cell solid-state NMR spectra. By integrating simulated ^13^C chemical shift maps with experimental ^13^C—^13^C and ^13^C—^1^H correlation spectra from ^13^C-enriched spruce, eucalyptus, and pine tissues, we define regions of unavoidable spectral overlap and identify diagnostic tannin correlations. While tannin A-ring signals extensively overlap with guaiacyl and syringyl lignin aromatics, repetitive B- and C-ring correlations provide unique spectroscopic fingerprints that enable reliable tannin identification across tissues. Application of these signatures reveals pronounced species- and tissue-dependent variation in tannin abundance and identifies conditions under which tannins dominate aromatic regions, complicating lignin analysis. More broadly, this work provides a transferable framework for distinguishing chemically similar aromatic polymers in complex solid-state NMR spectra and improves structural interpretation of intact plant biomass.

## Highlights

• Whole-cell ssNMR distinguishes condensed tannins from lignin in intact plant tissues.
• Diagnostic B-ring correlations provide robust spectral fingerprints of condensed tannins.
• Tannin A-ring signals extensively overlap with lignin aromatic resonances.
• Tannin abundance varies strongly among plant species and tissue types.
• The framework improves interpretation of whole-cell plant cell wall ssNMR spectra.

## Graphical abstract

**Figure:**
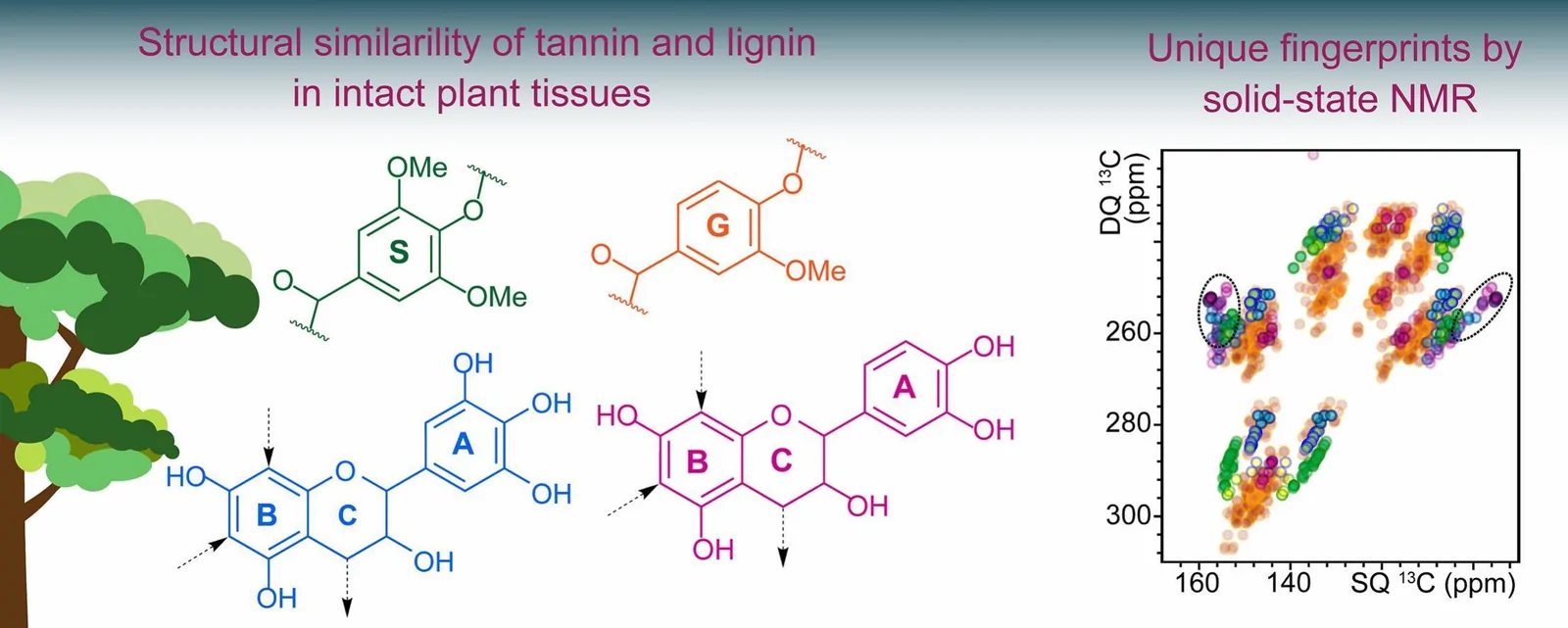

## 1 Introduction

Plant cell walls are structurally complex biological materials whose molecular organization underpins biomass recalcitrance, carbon cycling, and the development of renewable biomaterials (Debnath et al., 2025; Lankiewicz et al., 2023; Ragauskas et al., 2014). Solid-state NMR spectroscopy has emerged as a powerful technique for probing the architecture of native plant cell walls by simultaneously characterizing lignin, cellulose, and hemicelluloses without disrupting their native organization (Gil and Neto, 1999; Mao et al., 2006; Munson et al., 2022; Xiao et al., 2025a, Xiao et al., 2025b). Over the past decade, multidimensional solid-state NMR, often combined with isotopic labeling, advanced distance- and correlation-based experiments, and computational modeling, has shifted the field from compositional analysis toward architectural understanding, enabling polymer- and site-resolved models of how lignin associates with cellulose and hemicelluloses in intact secondary walls (Addison et al., 2024; Duan et al., 2021; Dupree et al., 2015). These studies have provided direct evidence for specific polysaccharide conformations and interfaces, such as xylan folding and packing on cellulose surfaces, and have mapped lignin-carbohydrate spatial proximities in situ (Gao et al., 2020; Kang et al., 2019; Kirui et al., 2022; Simmons et al., 2016; Terrett and Dupree, 2019; Terrett et al., 2019; Xiao et al., 2025a, Xiao et al., 2025b). However, as solid-state NMR is increasingly applied to intact and chemically complex tissues, whole-cell measurements face a fundamental analytical challenge: the coexistence of chemically similar aromatic polymers that generate overlapping spectral features.

Among the aromatic polymers present in plant tissues, condensed tannins represent a particularly important source of spectral congestion. Condensed tannins frequently coexist with lignin in bark, leaves, needles, and other tissues commonly examined by whole-cell solid-state NMR. Because their aromatic carbon environments closely resemble those of guaiacyl and syringyl lignin units, tannin-derived resonances extensively overlap with lignin signals, complicating spectral assignment and interpretation. Reliable identification of tannins is therefore essential for the accurate interpretation of lignin structure and lignin-carbohydrate interactions in intact biomass.

Although extraction- and separation-based analyses have established the composition and structural diversity of lignin and condensed tannins, these methods characterize the polymers after isolation from the plant cell wall (Fukushima and Hatfield, 2001; Rencoret et al., 2015; Vanholme et al., 2010; Vernhet et al., 2014). Differently, whole-cell solid-state NMR simultaneously detects lignin, tannins, and polysaccharides within intact tissues, where all components contribute to the same spectra (Li et al., 2018; Lorenz et al., 2000; Ma et al., 2018; Thompson and Pizzi, 1995; Ucar et al., 2013; Xue et al., 2024). Consequently, assigning aromatic resonances in whole-cell ssNMR requires analytical strategies that distinguish these chemically similar polymers directly in their native cellular context.

Condensed tannins, also known as proanthocyanidins, are ubiquitous secondary metabolites in vascular plants and are especially abundant in bark, leaves, and needles, where their concentrations can rival or sometimes exceed those of lignin (Crozier et al., 2006; Dixon and Sarnala, 2020; Heiska et al., 2007; Kumar, 1992; Moore et al., 2014; Pan et al., 2013). Condensed tannins contribute to plant defense, oxidative stress responses, and carbon cycling, and they influence biomass utilization by inhibiting enzymatic hydrolysis and complicating assessments of lignin abundance and reactivity in lignocellulosic feedstocks (Adamczyk, 2024; Barbehenn and Constabel, 2011; Crozier et al., 2006; Hagerman et al., 2025; Huang et al., 2024; Kumar, 1992; Mole and Waterman, 1987; Constabel et al., 2014; Tejirian and Xu, 2011). Their widespread occurrence in tissues frequently examined by whole-cell ssNMR further emphasize the need for reliable methods to identify and distinguish them in intact biomass (Navarrete et al., 2010; Newman and Porter, 1992; Seidi et al., 2025; Xia et al., 2001).

NMR spectroscopy provides complementary information on lignocellulosic biomass, ranging from solution-state characterization of lignin functional groups and interunit linkages to solid-state measurements of insoluble plant materials (Krivdin, 2025; Vuković and Tišma, 2024). Earlier work using specifically ^13^C-enriched lignin demonstrated the analytical capability of solid-state NMR for elucidating lignin structure in ginkgo wood (Terashima et al., 2002). The present study further addresses the discrimination of condensed-tannin and lignin contributions in whole-cell correlation spectra.

Here, we systematically analyze the ^13^C chemical shift signatures of condensed tannins and evaluate their overlap with lignin signals in whole-cell ssNMR spectra to establish experimentally validated spectral signatures for distinguishing these polymers in intact biomass. Using simulated tannin chemical shift maps alongside experimental data from spruce, eucalyptus, and pine tissues, we identify characteristic tannin spectral signatures that facilitate the differentiation of tannins from lignin. In particular, condensed tannins exhibit unique and highly repetitive correlations within their B-ring carbons that are absent in lignin, providing a basis for tannin identification in complex plant matrices. In addition, recent advances in fast magic-angle spinning (MAS) and ^1^H-detected ssNMR experiments provide enhanced spectral resolution and sensitivity, enabling complementary characterization of aromatic correlations in heterogeneous plant tissues. We further assess the implications of tannin interference for lignin-carbohydrate interaction analysis and provide practical guidelines for interpreting ssNMR spectra of tannin-rich plant materials.

## 2 Results and discussion

### 2.1 Structural basis and predicted NMR signatures of condensed tannins

To establish a structural and spectroscopic framework for tannin identification, we first summarize the chemical architecture of condensed tannins and their expected NMR signatures. Condensed tannins are polymers of flavan-3-ol units, primarily Pc and Pd, each comprising two aromatic rings (A and B) and one heterocyclic ring (Ring C) (Fig. 1A) (Hill et al., 2003; Nitao et al., 2001; Schofield et al., 2001). Polymerization occurs predominantly through C4-C8 interflavan linkages between B-ring carbons, with C4-C6 linkages occurring less frequently, typically associated with branching. These linkages give rise to terminal (T4*) and extension (T4β) units that are repeated along the tannin polymer backbone (Liu et al., 2021; Vera and Urbano, 2021).

**Fig. 1:**
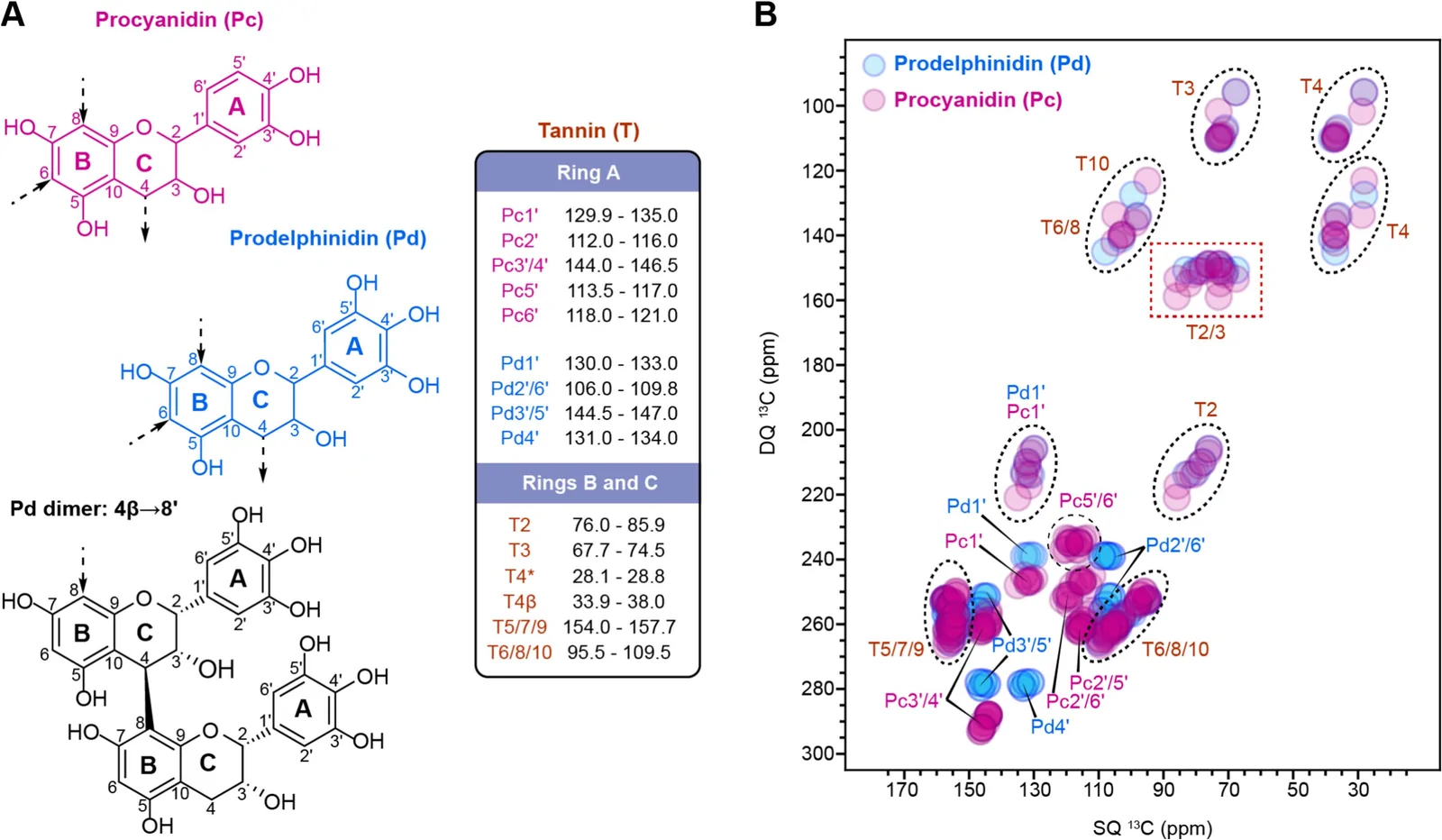
**Structure and chemical shift map of condensed tannins.** (**A**) The main structural units of condensed tannins (T) are colour-coded and mapped: the procyanidin (Pc) unit is shown in magenta and the prodelphinidin (Pd) unit in blue. Dashed arrows indicate inter-unit linkage positions. An example dimer featuring a 4–8 linkage between two Pd units is shown at the bottom. A table summarizing characteristic chemical shift ranges for signature tannin carbons is shown on the right. Carbon sites on ring A are denoted with apostrophes. Carbons of Rings B and C in Pc and Pd share similar chemical environments and are therefore labeled collectively as “T” rather than separately as Pc or Pd. T4* denotes the terminal unit carbon, whereas T4β represents the extension unit involved in 4–8 or 4–6 interflavan linkages. (**B**) Simulated 2D ^13^C spectrum of DQ-SQ correlations, generated from reported chemical shifts and predictions (Tables S1-S7). Each simulated peak is represented as a circle with 3 ppm diameter. Signature tannin fingerprint regions are enclosed with dashed lines. T2/3 region falls within carbohydrate peak regions (shown in **Fig. S2**) and is indicated brown dashed box. The T4-T6/8/10 regions may arise from both intramolecular (T4-T10) and intermolecular (T4-T8/T4-T6) correlations. (For interpretation of the references to colour in this figure legend, the reader is referred to the web version of this article.)

A major challenge in distinguishing tannins from lignin arises from the close structural resemblance between the tannin A-ring and lignin monolignols. In tannins, methoxy substituents present in lignin are replaced by hydroxyl groups, rendering the A-rings of Pc and Pd units homologous to G and S lignin units, respectively. As a consequence, the C1′ chemical shift ranges of both Pc and Pd units coincide with those of lignin aromatic carbons. In addition, the Pc C2′, C5′, and C6′ chemical shifts overlap extensively with those of most G-lignin units, while the Pd C4′ shifts coincide with typical S-lignin signals. Although Pd C3′/5′ carbons are generally more shielded than their S-lignin counterparts, these differences are often subtle relative to the broader statistical distributions of lignin signals in whole-cell spectra. Furthermore, despite the presence of a hydroxyl group at C3′ in Pc units, the Pc C3′/C4′ chemical shifts closely resemble those of certain G-lignin variants, leading to near-complete collapse of these peaks into a single aromatic cluster. Collectively, these similarities render tannin A-ring correlations largely indistinguishable from lignin aromatic signals in 1D ^13^C solid-state NMR experiments.

The INADEQUATE (Incredible Natural Abundance Double Quantum Transfer Experiment) experiments represent one of the most effective approaches for resolving aromatic spectral congestion and identifying spectral features unique to condensed tannins (Lesage et al., 1997; Lesage et al., 1999). By correlating the double-quantum (DQ) and single-quantum (SQ) chemical shifts of directly bonded carbon pairs in orthogonal dimensions, these experiments provide high spectral resolution, suppress diagonal signals, and enable unambiguous tracing of one-bond carbon‑carbon connectivities. We constructed a comprehensive DQ-SQ chemical shift map by simulating one-bond correlations from reported experimental chemical shifts and predictive calculations (Fig. 1B). The resulting analysis reveals that the most definitive tannin fingerprints arise from B-ring correlations, particularly the T5/7/9-T6/8/10 cross-peaks. These correlations appear at approximately 154.0–157.7 ppm (T5/7/9) and 95.5–109.5 ppm (T6/8/10), regions that are well separated from all known lignin aromatic carbon shifts. Notably, these are among the strongest tannin signals, as each resonance represents three chemically similar carbons that are repeatedly present along the polymer chain.

Additional, secondary tannin fingerprints are predicted near the carbohydrate region, including T4-T6/8/10 and T4-T3 correlations (**Fig. S2**). Although the SQ chemical shifts of T3 and T6/8/10 overlap with those of many carbohydrate carbons, the T4 carbon occupies distinct chemical shift ranges, approximately 33–38 ppm for extension units (T4β) and 2–29 ppm for terminal units (T4*), allowing these correlations to be distinguished from polysaccharide signals. A further semi-unique tannin signature is provided by the C1′-T2 correlation linking the A- and B-rings. While the C1’ shifts overlap with lignin aromatic carbons, the T2 chemical shifts (76–86 ppm) are generally higher than those of the most abundant lignin Cα carbons, enabling this correlation to serve as a complementary indicator of tannin presence when observed alongside primary B-ring fingerprints. Despite their participation in characteristic tannin correlations, T2 and T3 participate in characteristic tannin correlations, their chemical shifts overlap extensively with carbohydrate resonances in the 60–90 ppm region (**Fig. S2**), limiting their utility as standalone diagnostic markers.

### 2.2 Spectral overlap and separability of tannin and lignin aromatic signals

To quantitatively evaluate the separability of condensed tannin and lignin signals in the aromatic region, the simulated tannin chemical shift map was overlaid with comprehensive chemical shift distributions for all known guaiacyl (Fig. 2A) and syringyl (Fig. 2B) lignin variants (Ralph et al., 2004a, Ralph et al., 2004b). This comparison demonstrates that the vast majority of tannin A-ring chemical shifts coincide extensively with lignin aromatic carbons, resulting in near-complete spectral overlap across much of the aromatic region, even in the DQ-SQ ^13^C correlation spectra. Consequently, tannin and lignin signals are effectively indistinguishable in conventional SQ-SQ ^13^C—^13^C correlation spectra.

**Fig. 2:**
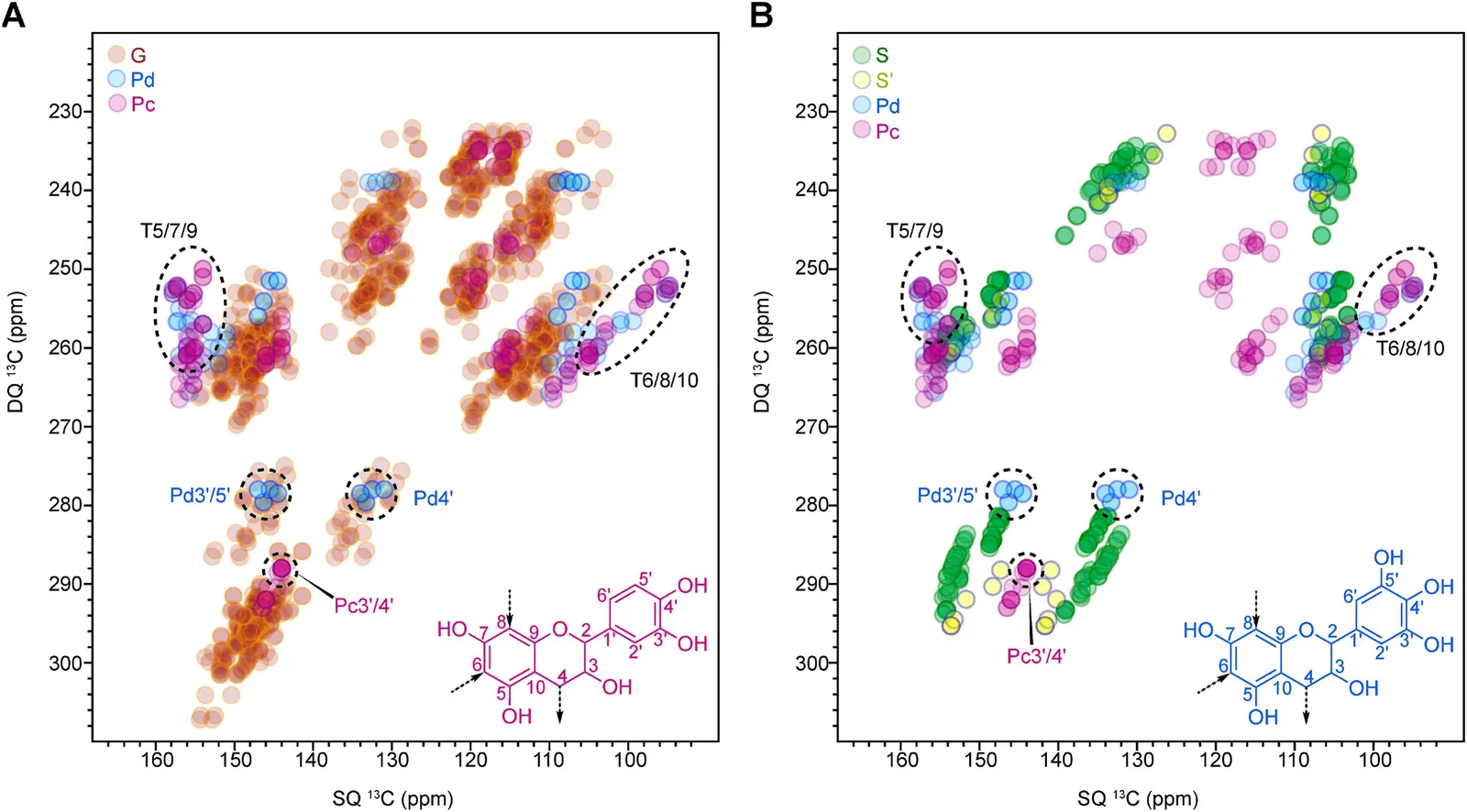
**Comparison of tannin and lignin aromatic signals in DQ-SQ**^**13**^**C spectra.** Simulated DQ-SQ chemical shift maps of condensed tannins are overlaid with comprehensive lignin chemical shift distributions for (**A**) all guaiacyl lignin variants (brown) and (**B**) all syringyl lignin variants (green) and Ca oxidized S units (yellow). The structure of two main tannin units are given in each panel, with the carbon site labelling system following (Fig. 1**)**. The tannin peak regions that can be notably distinguished from lignin regions are enclosed in dashed lines. Extensive overlap is observed between tannin A-ring and lignin aromatic chemical shifts in both G- and S-rich regions. In contrast, the tannin B-ring T5/7/9-T6/8/10 correlations (dashed circles) occupy regions that are separated from all lignin aromatic variants, constituting unambiguous spectral fingerprints for condensed tannins. (For interpretation of the references to colour in this figure legend, the reader is referred to the web version of this article.)

Despite this pervasive overlap, the analysis reveals a single class of unambiguous tannin signatures in the aromatic region: the B-ring T5/7/9-T6/8/10 correlations (dashed circles in Fig. 2A, B). These cross-peaks occupy narrow and well-defined chemical shift ranges that are completely separated from all lignin aromatic variants, making their presence a definitive indicator of condensed tannins in solid-state NMR spectra of these whole-cell samples. Detection of this peak pair therefore serves as a necessary confirmation of tannin content in a given sample.

In addition to these primary fingerprints, the A-ring Pc3′-Pc4′ and Pd3′/5′-Pd4′ correlation patterns retain diagnostic value under favorable conditions. When tannin signals are sufficiently intense relative to lignin, these correlations can be used to differentiate and estimate the relative contributions of Pc and Pd units. However, because these peak patterns fall within the broader distributions of G- and S-lignin aromatic correlations, their practical utility is contingent on tannin abundance and spectral dominance.

Importantly, the extensive overlap of tannin A-ring and lignin aromatic chemical shifts implies that once the presence of condensed tannins is confirmed by the T5/7/9-T6/8/10 fingerprint in a DQSQ spectrum, all aromatic cross-peaks must be interpreted with caution. The substantial overlap between tannin and lignin aromatic resonances in one-dimensional ^13^C spectra is illustrated in **Fig. S1**, highlighting the need for multidimensional correlation experiments to achieve reliable spectral discrimination. In tannin-rich samples, aromatic correlations cannot be assumed to originate exclusively from lignin, which explicitly necessitates tannin identification prior to lignin assignment and quantitative analysis.

### 2.3 Identification of condensed tannins in spruce and eucalyptus tissues

We next applied the tannin chemical shift map to experimental spectra acquired from whole-cell spruce and eucalyptus stem samples to evaluate tannin detectability in real-life plant tissues (Fig. 3). In spruce, the presence of condensed tannins is clearly established by strong and well-resolved B-ring fingerprint correlations, most notably the T5/7/9-T6/8/10 peak pairs observed at DQ of 253 ppm with corresponding SQ pair of 155 ppm and 98 ppm, as well as at DQ of 261 ppm with corresponding SQ pair of 155 ppm and 98 ppm (Fig. 3A, dashed lines). These correlations occupy spectral regions that are completely separated from all guaiacyl lignin variants, providing unambiguous confirmation of tannin presence. This assignment is further supported by detection of the C1′-T2 correlation linking the A- and B-rings (Fig. 3A, dashed box).

**Fig. 3:**
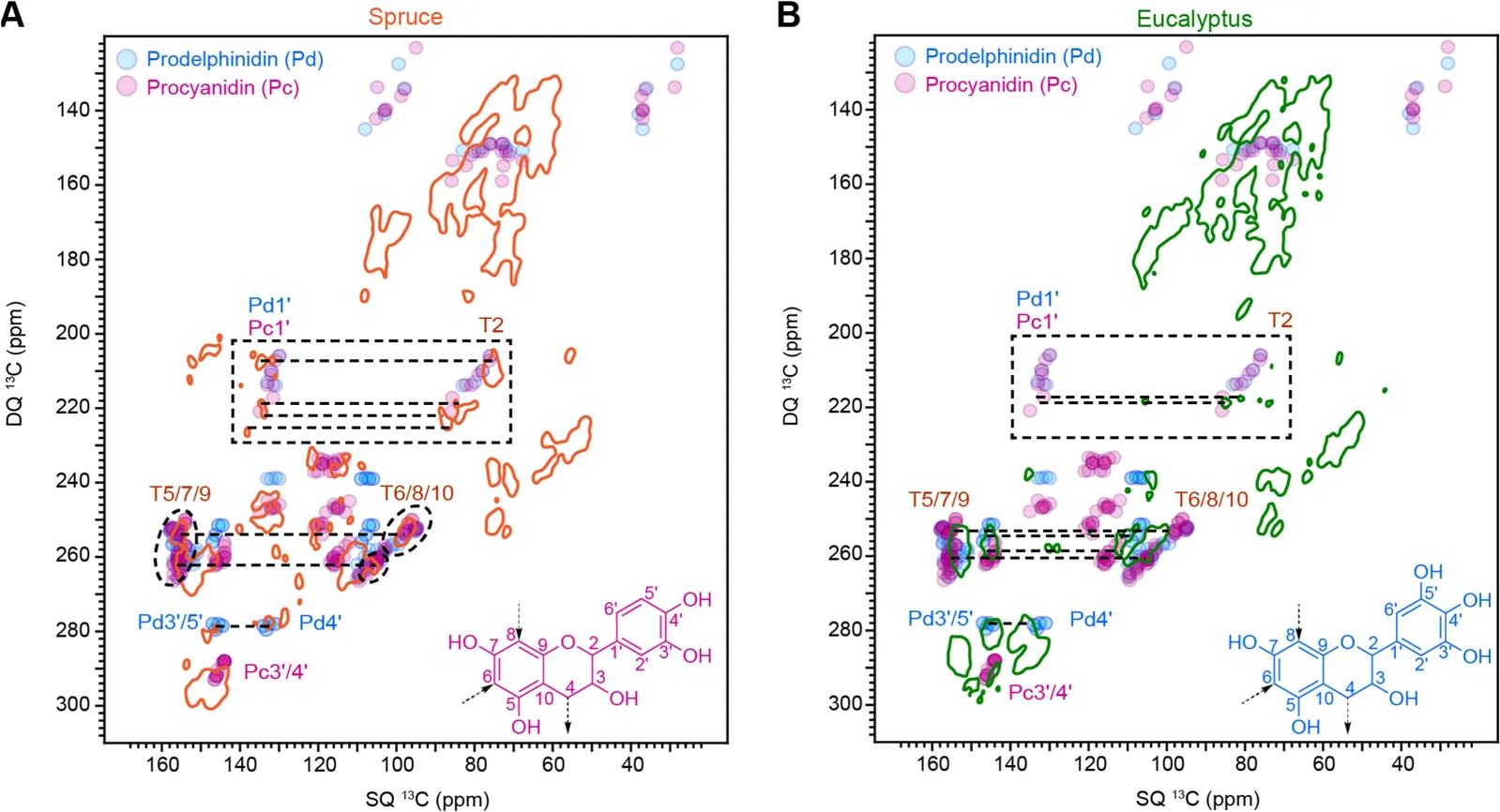
**Identification of condensed tannins in spruce and eucalyptus by solid-state NMR.** Full tannin DQ-SQ chemical shift maps are overlaid with experimental spectra from (**A**) spruce and (**B**) eucalyptus. The experimental spectra cutoff level is at 4.5 standard deviations (sigma) for spruce and 5 sigma for eucalyptus, with only the first base contour of experimental spectra displayed. In spruce, strong and well-resolved T5/7/9-T6/8/10 B-ring correlations are enclosed by dashed lines and T2-C1′ region is highlighted in dashed box, with their connectivities indicated with horizontal dashed lines. In eucalyptus, potential T2-C1′ (enclosed in dashed box) and T5/7/9-T6/8/10 peak positions are indicated by horizontal dashed lines.

Additional predicted tannin correlations near the carbohydrate region, such as T4-T10 (DQ of 138 ppm, with SQ pair of 38 ppm and 100 ppm), are not detected in this spruce spectrum, likely reflecting limitations in experimental sensitivity and optimization. Although, in principle, differentiation between Pc and Pd units could be achieved through the Pc3′-Pc4′ and Pd3′/5′-Pd4′ correlation patterns, these peaks are obscured by the dominant G-lignin aromatic signals in spruce, precluding reliable discrimination of Pc and Pd contributions.

In contrast, the presence of tannin in the eucalyptus sample is possible but not convincing (Fig. 3B). The tannin fingerprint regions only marginally intersect with the spectral contours, and the characteristic T5/7/9-T6/8/10 correlations are weak and poorly resolved. The C1′-T2 correlation is also faint, and peaks in this region could alternatively be assigned to lignin C1-Cα correlations. Similarly, the Pd3′/5′-Pd4′ and Pc3′-Pc4′ correlation maps only minimally overlap with the eucalyptus spectra and are more plausibly attributed to G-lignin aromatic signals. Taken together, these observations suggest that condensed tannins are either present only at low abundance in this eucalyptus sample or are spectroscopically obscured by overlapping lignin signals.

An alternative explanation for a subset of the unassigned aromatic correlations observed in eucalyptus is the presence of hydrolyzable tannin-derived structures, such as gallic acid or tannic acid. Predicted chemical shifts for these compounds partially overlap with the experimental spectra, particularly near DQ ∼256 ppm (Fig. S4). However, the overall agreement is limited. In particular, the strongest experimental correlation near DQ ∼280 ppm and the characteristic C1-C7 carbonyl-aromatic correlation are not reproduced by the predicted gallic acid or tannic acid chemical shifts. Moreover, hydrolyzable tannins are generally considered extractable soluble metabolites rather than major structural constituents of plant cell walls (Hernes and Hedges, 2000, Hernes and Hedges, 2004; Lu et al., 2007). Collectively, these observations suggest that hydrolyzable tannin-derived structures may contribute to a subset of the observed signals but do not provide sufficient evidence for their unambiguous assignment.

### 2.4 Impact of tannins on lignin and carbohydrate analysis in spruce

To evaluate how condensed tannins affect lignin- and carbohydrate-focused solid-state NMR analyses in softwood tissues, we examined spruce stem tissue using a combination of ^13^C- and ^1^H-detected experiments with varying polarization transfer conditions. We first employed the same ^13^C refocused J-INADEQUATE experiment to establish carbon‑carbon connectivities and to disentangle overlapping aromatic signals from lignin and tannins (Fig. 4A). The inclusion of a DQ dimension substantially enhances spectral dispersion, enabling clear differentiation of tannin-derived ^13^C connectivities from those of guaiacyl lignin units, which dominate the spruce lignin composition.

**Fig. 4:**
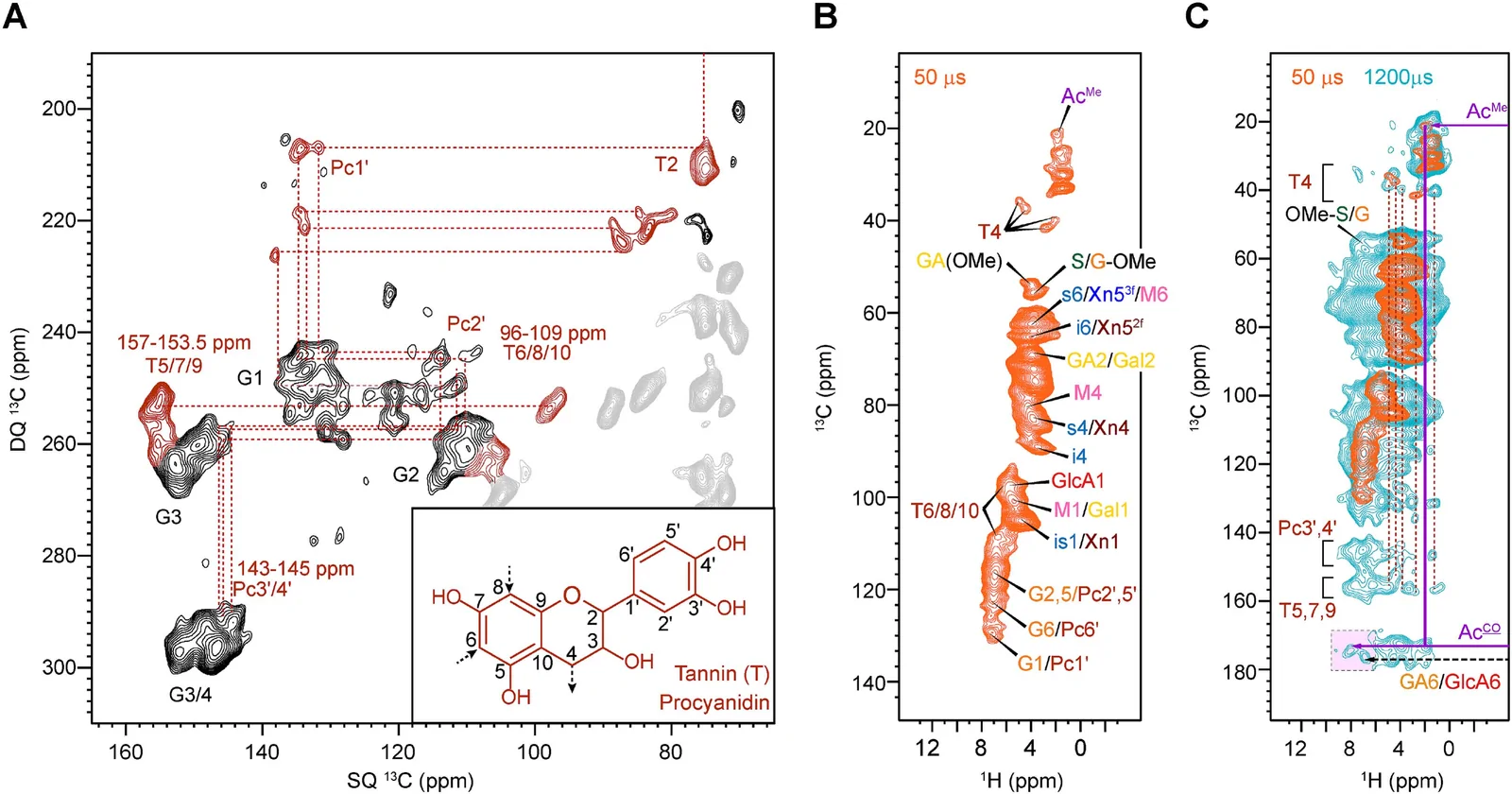
**Analysis of tannin, lignin, and carbohydrate components in spruce via**^**13**^**C and**^**1**^**H-detection.** (**A**) 2D CP ^13^C refocused *J*-INADEQUATE spectrum of spruce showing signals of G-lignin (black) and tannin (brown). The connectivities of tannin carbons are indicated by dashed lines. Dominant carbohydrate-derived correlations are displayed in low-opacity gray for visual clarity and to highlight weaker tannin-associated correlations. MAS spinning sideband (SSB) from intense carbohydrate region are in gray. Contour features in the SQ 60–80 ppm and DQ 220–240 ppm region originate primarily from carbohydrate (GalA/GlcA5) correlations and associated spinning sidebands. Signature tannin peaks can be identified for T5/7/9 at SQ chemical shift of 153–157 ppm, T6/8/10 at 96–109 ppm, and potential Pc3′/4′ at 143–145 ppm. (**B**) 2D hCH spectrum of spruce with a short second ^13^C—^1^H CP contact time of 50 μs showing mostly one-bond correlations. (**C**) Overlay of two 2D hCH spectra with short (orange, 50 μs) and long (blue, 1.2 ms) ^13^C—^1^H CP contact times. Many tannin intramolecular cross-peaks are observed and are indicated with brown vertical dashed lines. Carbohydrate-lignin cross-peaks are highlight in purple box. Connectivity of acetyl signals are highlighted using the purple lines. (For interpretation of the references to colour in this figure legend, the reader is referred to the web version of this article.)

Guided by the ^13^C-based assignments, we then analyzed a ^1^H-detected hCH spectrum acquired with a short second ^13^C—^1^H cross-polarization (CP) contact time of 50 μs (Fig. 4B). The ^1^H-detected solid-state NMR experiments enabled by MAS (typically 60–110 kHz) were originally developed for protein studies and more recently extended to carbohydrates and biomass (Duan and Hong, 2024; Le Marchand et al., 2022; Lends et al., 2025; Xiao et al., 2025c; Yarava et al., 2025; Yarava et al., 2025), providing fast acquisition, enhanced sensitivity and additional nucleus dimension with decent spectral resolution. Unfortunately, we encountered substantial spectral overlap between tannin-derived signals and G-lignin aromatics in two-dimensional ^13^C—^1^H correlations. Despite this overlap, several tannin resonances could be confidently identified, most notably at the C4/H4 (T4) position, the key interflavan linkage site, where four distinct environments were resolved at approximately (35.8, 4.95 ppm), (37.3, 4.4 ppm), (39.9, 2.0 ppm), and (41.4, 2.7 ppm), reflecting the structural heterogeneity of condensed tannins.

Additional tannin signals, including those from the T6/8/10 carbons, were also distinguishable from carbohydrate and lignin resonances. The short CP spectrum further reveals rigid-domain signals from two- and three-fold xylan (Xn) conformations, internal and surface cellulose chains (i and s), and mannose residues (M) from galactoglucomannan (GGM), a hemicellulose enriched in softwoods (Fig. 4B). Acetyl group resonances (Ac^Me^) are also observed; unlike in eucalyptus, these acetylations are associated primarily with GGM in spruce secondary cell walls (Hannuksela and du Penhoat, 2004; Xu et al., 2010). Although spruce contains arabinoglucuronoxylan (AGX) with arabinose and glucuronic acid (GlcA) substitutions (Scheller and Ulvskov, 2010; Terrett and Dupree, 2019), arabinose residues remain largely invisible under these CP-based conditions due to their high mobility (Kirui et al., 2022).

Extending the second CP contact time from 50 μs to 1.2 ms enhances long-range magnetization transfer, revealing additional intermolecular correlations (Fig. 4C). Under these conditions, tannin carbons exhibit strong cross-peaks to their own aliphatic and hydroxyl protons, producing pronounced signal enhancement in the T3′/4′ and T5/7/9 regions. Consequently, tannin resonances dominate the aromatic region, complicating analyses of lignin-carbohydrate interactions that rely on aromatic carbons as starting points. Nevertheless, clear intermolecular correlations between acetyl carbonyl (Ac^CO^) and GA6/GlcA6 carbon sites and aromatic protons remain detectable (Fig. 4C, purple box), demonstrating that selected carbohydrate-aromatic interactions can still be resolved despite extensive magnetization transfer.

The enhanced ^1^H resolution afforded by fast-MAS ^13^C—^1^H correlation experiments provides valuable complementary assignment information. However, because aromatic lignin and tannin regions are relatively proton deficient, the utility of ^1^H—^1^H correlation experiments for resolving these components remains limited. Instead, the present results highlight the potential of higher-dimensional ssNMR experiments, such as 3D DQ-SQ-SQ spectroscopy incorporating an additional ^13^C DQ dimension, to further improve spectral resolution and facilitate analysis of spatial proximities in tannin-rich plant tissues (Reddy et al., 2016; Shekar et al., 2022).

The spectral signatures established here complement existing solid-state NMR and chemical approaches for lignin and tannin characterization. Combined with multidimensional correlation experiments, isotopic labeling, and complementary chemical analyses, they provide a framework for investigating tannin-rich plant cell walls. Natural-abundance solid-state NMR provides established approaches for biomass characterization, including one-dimensional experiments, spectral editing, and selected multidimensional experiments (Addison et al., 2025). The sensitivity demands are greater for the ^13^C—^13^C correlation measurements used here, which benefit substantially from isotopic enrichment. DNP offers an additional route to acquiring such correlations at natural abundance. Our implementation therefore targets detailed structural investigations using ^13^C-enriched tissues; its extension to unlabeled biomass would require assessment of sensitivity, resolution, and acquisition time. Consequently, this methodology is best suited for detailed structural investigations rather than routine analytical measurements.

### 2.5 Tissue-dependent distribution of condensed tannins in pine

To assess tissue-dependent variability in tannin abundance and further validate the robustness of the tannin fingerprints, we analyzed the INADEQUATE spectra collected on multiple pine tissues, including bark, needle, root, and stem. Full tannin connectivity is clearly observed in pine bark and, to a lesser extent, in needle and root tissues (Fig. 5A-C**, Fig. S2**). In contrast, stem samples exhibit only guaiacyl lignin signals and no detectable tannin correlations (Fig. 5D), indicating effective debarking and confirming that the stem material is free of tannin interference.

**Fig. 5:**
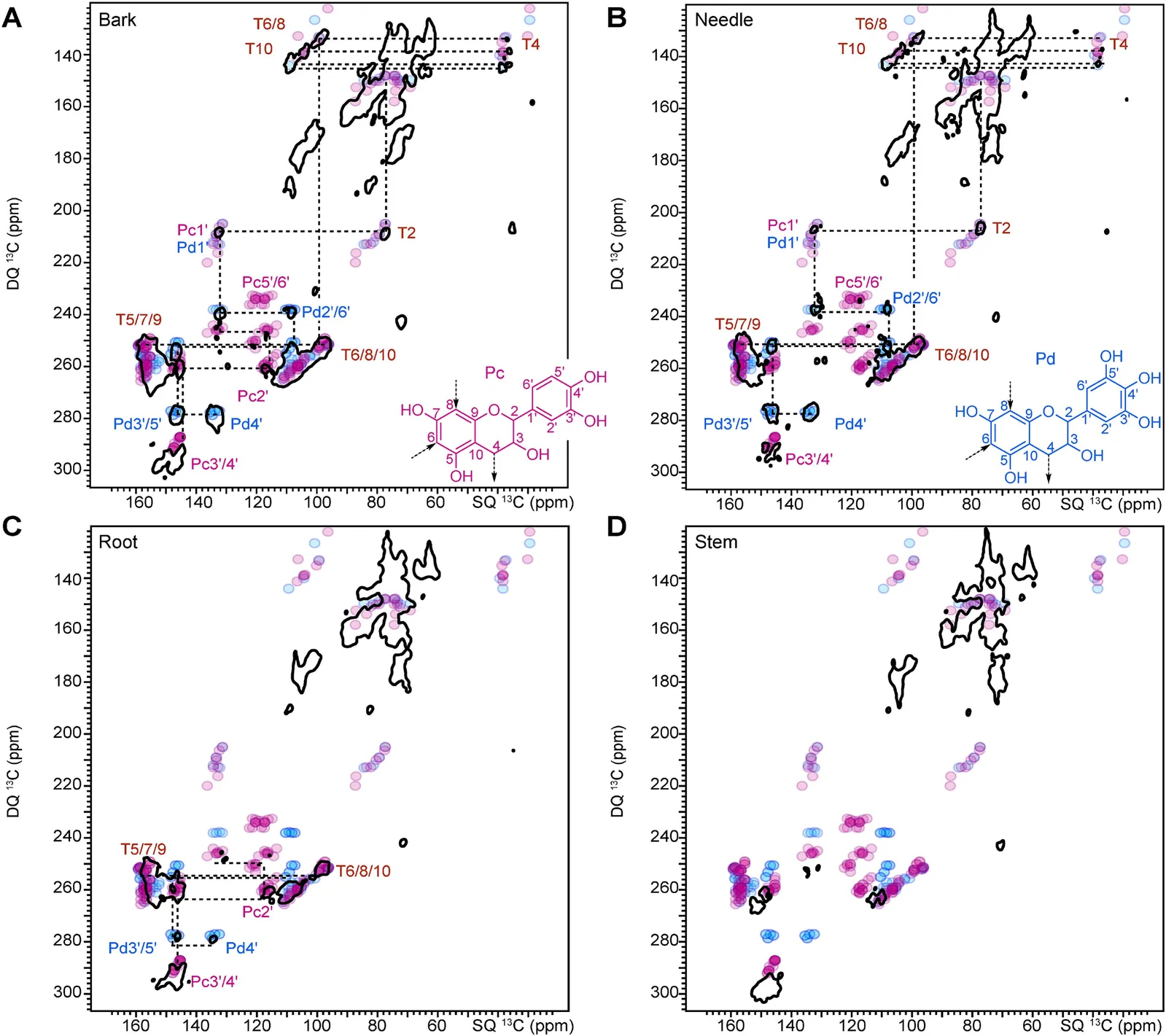
**Tissue-dependent identification of condensed tannins in pine by solid-state NMR.** Full tannin chemical shift maps are overlaid with experimental INADEQUATE spectra from pine (**A**) bark, (**B**) needle, (**C**) root, and (**D**) stem tissues. Only the first base contour of each plant spectrum is shown, with the base cut-off level at 4.5–5 sigma. Structural representations of the identified tannin units are displayed within each spectrum. Signature tannin correlations are indicated by black dashed lines. In root and stem samples, the absence of detectable tannin signals is evident from the lack of corresponding correlations, e.g. T4-T6/8/10, observed in bark and needle tissues.

Among the analyzed tissues, pine bark shows the most complete set of tannin correlations, including primary B-ring fingerprints and secondary connectivities, consistent with high tannin abundance (Fig. 5A). Selected SQ-dimension cross-sections extracted from representative DQ positions in the pine bark DQ-SQ spectrum are shown in **Fig. S3**. These cross-sections demonstrate the spectral resolution of the key tannin and lignin correlations and reveal signal-to-noise ratios of 86–89 for Pd3′/5′ and Pd4′, 30–55 for T5/7/9 and T6/8/10, and 7–10 for the lignin G3/4 correlation, highlighting the dominant contribution of tannin signals in the aromatic region. Additional correlations such as T3-T4 are present in the bark spectrum and are only displayed in **Fig. S2** due to limitations in spectral windowing. Recently, solid-state NMR has been applied to the quantitative and structural characterization of native lignin in hardwood and softwood bark (Xue et al., 2024). Our results on pine bark complement this work by demonstrating a substantial contribution of condensed tannin to the aromatic region of the spectra. Identifying these contributions is therefore an important step in selecting and interpreting aromatic signals for analysis of lignin and its interaction with carbohydrate partners in tannin-rich bark. The present measurements establish assignments qualitatively and do not independently quantify lignin or tannin content.

Needle samples also exhibit strong tannin fingerprints, but partial truncation of the spectral window, particularly at lower DQ values below 120 ppm, prevents visualization of the full tannin connectivity (Fig. 5B). Extending the lower limit of the DQ dimension to approximately ∼100 ppm would be required to capture all tannin-related correlations in these samples (**Fig. S2**). Root tissues display weaker but still discernible tannin signals, indicating reduced tannin content relative to bark and needle (Fig. 5C).

The tissue-dependent spectral differences observed in Fig. 5 are consistent with the known enrichment of condensed tannins in bark and needles relative to stem tissues (Hernes and Hedges, 2004; Hussein et al., 1990; Kwan et al., 2022; Magina et al., 2024; Sartori, 2018; Zhang et al., 2017). Analysis of unit-specific correlations further suggests that prodelphinidin is the dominant tannin unit in pine tissues. Characteristic Pd3′/5′-Pd4′ correlations are most prominent in bark and needle samples and decrease in intensity in root tissues, consistent with a declining Pd contribution (Fig. 5A-C). Together with the absence of detectable tannin signals in stem tissue, these results demonstrate pronounced tissue-dependent variation in tannin distribution within pine.

More generally, condensed tannins can be reliably identified and distinguished from lignin by their characteristic fingerprint correlations, including the T5/7/9-T6/8/10 B-ring cross-peaks and the Pd3′/5′-Pd4′ and Pc3′-Pc4′ correlation patterns. When present, these signatures also provide a basis for estimating the relative contributions of tannin and lignin signals in the aromatic region of whole-cell solid-state NMR spectra.

Studies of lignin-biosynthesis genes and tannin-containing seed walls in Arabidopsis highlight the potential of this model system for investigating aromatic components in plants (Chun et al., 2019; Demonsais et al., 2020). Future comparisons of appropriate tissues from wild-type plants and well-characterized lignin- or tannin-biosynthesis mutants, supported by complementary chemical analyses, would provide independent tests of the aromatic assignments established here. The occurrence of tannins within plant cell walls has experimental precedent. Tannin-containing walls have been identified in Arabidopsis seed tissues, while histochemical and fluorescence studies have demonstrated wall-associated condensed tannins in roots of *Pinus banksiana*, *Eucalyptus pilularis*, and Populus species (Chowdhury et al., 2023; Demonsais et al., 2020; McKenzie and Peterson, 1995). These observations provide a biological basis for investigating tannins as wall-associated components, but their localization cannot be transferred directly to the species, tissues, and developmental stages examined here. The present NMR data establishes diagnostic tannin correlations without resolving their cellular distribution or specific associations with other wall polymers. Combining spatially resolved tannin localization with additional solid-state NMR experiments would help distinguish intracellular and wall-associated populations and constrain their molecular organization. Such complementary evidence is needed to develop structural models that extend beyond the component identification established in this study.

Extraction-based methods, including HPLC and colorimetric assays such as Folin-Ciocalteu and butanol-HCl, remain highly effective for quantifying condensed tannins. In contrast, the solid-state NMR approach developed here enables direct observation of lignin and condensed tannins within intact plant tissues while preserving their native cellular context and simultaneously correlating their signals with other cell wall components. Although the present study is qualitative, the experimentally validated spectral markers established here provide a foundation for future efforts to quantify condensed tannins and investigate tannin-cell wall interactions directly in intact biomass using solid-state NMR. Quantitative ^31^P NMR following phosphitylation provides complementary measurements of hydroxyl and carboxylic acid functionalities in dissolved lignin and tannin samples (Argyropoulos et al., 2021). Benchtop ^31^P NMR has also been evaluated for quantitative lignin characterization through comparison with high-field measurements (Araneda et al., 2022). These approaches require sample dissolution and derivatization, whereas the present solid-state measurements identify tannin-associated carbon connectivities directly in unextracted tissues. Combining these methods could connect functional-group measurements with assignments in intact biomass, provided that extraction recovery and sample representativeness are evaluated.

## 3 Conclusions

This study addresses an analytical challenge in whole-cell solid-state NMR by establishing experimentally validated spectral criteria for distinguishing condensed tannins from lignin in intact plant tissues. The resulting spectral assignments and correlation maps provide a framework for future solid-state NMR investigations of tannin-rich biomass, including studies of lignin-tannin interactions, cell wall organization, and biomass deconstruction. Our analysis demonstrates that tannin interference is an intrinsic consequence of the structural similarity between tannin A-rings and lignin aromatics, explaining why lignin assignments based solely on aromatic correlations can be ambiguous. In contrast, repetitive B-ring correlations provide robust and transferable spectral fingerprints for condensed tannins. We further show that tannin interference is highly context dependent, varying substantially among species and tissues, and can constrain aromatic-based analyses of lignin-carbohydrate interactions under commonly used polarization-transfer conditions. Conversely, the absence of tannin signatures in selected tissues confirms that whole-cell solid-state NMR can provide unambiguous lignin information when tannins are absent or properly identified. Beyond improving spectral assignments, the framework established here provides practical guidance for experimental design and interpretation of whole-cell ssNMR data from chemically complex plant tissues. More broadly, this work expands the analytical capability of solid-state NMR for intact biomass and establishes a foundation for future multidimensional approaches for resolving chemically similar polymers in complex biological materials.

## 4 Materials and methods

### 4.1 ^13^C-labeling plant tissues

Uniformly ^13^C-enriched mature stems of *Eucalyptus grandis* and *Picea abies* were produced by growth in ^13^CO_2_-enriched (97 atom%) closed-atmosphere chambers at IsoLife (Wageningen, The Netherlands) for 16 weeks. Following harvest, stems were debarked, finely sectioned using a razor blade, and directly packed into 1.3 mm MAS rotors for solid-state NMR analysis (Xiao et al., 2025c, Yarava et al., 2025). For *Pinus radiata*, small stem cuttings (∼5 cm) were generated from two-year-old trees by Kools Sierteeltkwekerij (Deurne, The Netherlands). Cuttings were planted in 9 × 9 cm pots containing a 1:1:1 (*v*/v/v) mixture of vermiculite, perlite, and rockwool. After 7 months of growth, callus tissue had developed, and the plantlets were transferred to IsoLife for isotopic enrichment. Labeling was performed for 6 months in an atmosphere containing 99 atom% ^13^CO_2._ Following labeling, *P. radiata* plantlets (10–15 cm in height) were harvested. Stems were debarked, and needles and roots were separated. Individual tissues (stem, bark, needle, and root) were flash-frozen, freeze-dried, and subsequently analyzed by solid-state NMR (Cresswell et al., 2025; Cresswell et al., 2021).

### 4.2 Solid-state NMR analysis experiments

All solid-state experiments were acquired on a Bruker Avance NEO 600 MHz (14.1 T) spectrometer. Solid-state NMR experiments were performed on a Bruker Avance NEO 600 MHz (14.1 T) spectrometer equipped with a 1.3 mm HCN MAS probe operating at 60 kHz MAS. ^13^C chemical shifts were externally referenced to tetramethylsilane (TMS) via adamantane (38.48 ppm), and ^1^H shifts were referenced to DSS (0 ppm). Two-dimensional ^1^H-detected hCH spectra were collected using a 1.3 mm HCN MAS probe operating at 60 kHz MAS rate with bearing gas temperature set to 250 K (corresponding to an estimated sample temperature of ∼296 K), and ^13^C-detected spectra were collected using a 3.2 mm HCN triple-resonance probe operated under 14–18 kHz MAS rate at sample temperatures ranging 275–294 K. The bearing gas temperature was set to 250 K, corresponding to an estimated sample temperature of ∼296 K under fast MAS. All spectra were acquired using Cross-Polarization (CP) (Pines et al., 1973; Urbina and Waugh, 1974)-based method and detect only the rigid domains of native cell. For the ^1^H-detected hCH experiments, an initial 1.2 ms ^1^H—^13^C CP was used to excite all ^13^C signals, followed by either a 50 μs ^13^C—^1^H CP for short-range correlations (mostly direct ^13^C—^1^H pairs) or a 1.2 ms ^13^C—^1^H CP for long-range correlations. In hCH experiments, uppercase letters denote the nuclei that were measured and the lower case “h” denotes that the initial ^1^H frequency evolution is not measured. For the Two-dimensional ^13^C—^13^C refocused *J*-INADEQUATE (Lesage et al., 1997; Lesage et al., 1999) spectra were acquired on a 600 MHz spectrometer using a 3.2 mm MAS probe at 14 kHz MAS with a, the *J*-evolution period τ (half of the spin echo) was set to 1.4–1.5 ms to maximize the DQ coherence conversion. Delay (τ) of 1.42 ms to optimize one-bond correlations, the sample temperature was ∼294 K. Radiofrequency field strengths were 83 kHz for ^1^H decoupling, with 90° pulse rf fields of 83.3 kHz (^1^H) and 62.5 kHz (^13^C). Short-range ^1^H—^13^C correlations were obtained using 2D hCH experiments with a short CP contact time (50 μs) for direct C—H pairs and a longer contact time (1.2 ms, spruce) to enhance rigid aromatic signals. For the fast-MAS ^1^H-detected experiments, less than 3 mg of sample was packed into each 1.3 mm rotor. For the 3.2 mm MAS experiments, approximately 50 mg of pine tissue was packed into each rotor. Representative acquisition times were approximately 1 h 49 min for the 2D ^13^C—^1^H correlation experiments, ∼10 h for the spruce refocused *J*-INADEQUATE spectrum, ∼7 h for the eucalyptus refocused *J*-INADEQUATE spectrum, and 13 h 30 min for the pine refocused *J*-INADEQUATE spectrum. Detailed experimental parameters for all 2D NMR experiments are provided in **Supplementary Table S1**. All experimental spectra are displayed with a base cut-off level at 4.5–5 fold of the noise level (sigma). For Fig. 3, Fig. 5, only one contour level (base cut-off level) was displayed for simplicity; for Fig. 4, 32 levels of contours were displayed with a multiplication factor of 1.15.

### 4.3 Chemical shift mapping and generation of simulated spectra

A comprehensive lignin chemical shift reference dataset was compiled by integrating information from published solution-state NMR data, published solid-state NMR data, and ChemDraw-predicted chemical shifts, with all values referenced to tetramethylsilane (TMS). The carbon chemical shifts, solvent conditions, monolignol unit types, and linkage assignments used for spectral simulation are summarized in **Tables S2-S9**.

For ChemDraw predicted data, the solvent was set to DMSO. For solution NMR data, we chose the most biologically relevant dimers and trimers, i.e. only the model compounds with their linkages and aliphatic sites reported in real plants were included, from the database compiled by Ralph and colleagues (Ralph et al., 2004a, Ralph et al., 2004b). For each model compound, chemical shift datasets acquired in both dimethyl sulfoxide (DMSO) and acetone were included to account for solvent-dependent variations. Although solid-state NMR chemical shifts may differ from their solution-state counterparts because of differences in molecular conformation and local environment, the solution-state values provide a reasonable approximation for the present analysis. This assumption is supported by the strong agreement between the simulated peak distributions and the experimental spectra, as well as by recent high-resolution solid-state NMR studies of secondary plant cell walls that report comparable chemical shift values.

The simulated DQ-SQ peaks from reference **Tables S2-S9** were generated via homemade Python scripts. Each peak is represented by a circle with 3 ppm diameter to mimic the intrinsic broad linewidth observed in whole cell. The generated peaks were overlaid with experimental spectra plotted using a Python 3 package “NMR Penguins”. The NMR Penguins package is available on Github: https://github.com/yongrenjie/penguins for public access.

## Associated content

Additional solid-state NMR data and analysis are provided in the Supporting Information, which includes **Figs. S1**-**S4** and **Tables S1-S9** and supporting references.

## CRediT authorship contribution statement

**Priya Sahu:** Writing – original draft, Investigation, Formal analysis. **Peng Xiao:** Writing – original draft, Investigation, Formal analysis. **Debkumar Debnath:** Writing – review & editing, Formal analysis. **Jayasubba Reddy Yarava:** Writing – review & editing, Investigation. **Qinghui Cheng:** Writing – review & editing, Formal analysis. **Tuo Wang:** Writing – review & editing, Conceptualization.

## Funding

This work was supported by the 10.13039/501100004477US 10.13039/100000015Department of Energy (DOE), 10.13039/100006132Office of Science, 10.13039/100006151Basic Energy Sciences, under award number DE-SC0023702.

## Declaration of competing interest

The authors declare the following financial interests/personal relationships which may be considered as potential competing interests: This work was supported by the US Department of Energy (DOE), Office of Science, Basic Energy Sciences, under award number DE-SC0023702 to Tuo Wang.

## Data Availability

The complete simulation script package including the CSV format of the tables and raw NMR data was deposited in Zenodo, under DOI: https://doi.org/10.5281/zenodo.22762215 for public access.
