# Supplementary material for: Resolving aromatic spectral congestion in whole-cell solid-state NMR: Signatures of condensed tannins and lignin in plant tissues
Source: Cell Surf. 2026 Sep 22;16:100182. doi: 10.1016/j.tcsw.2026.100182 (PMC13634393; doi:10.1016/j.tcsw.2026.100182)
Supplement: Supplementary file 1 [file mmc1.pdf]

Supporting Information

Resolving Aromatic Spectral Congestion in Whole-Cell Solid-State NMR: Signatures of Condensed Tannins and Lignin in Plant Tissues

Priya Sahu[1#], Peng Xiao[1#], Debkumar Debnath[1], Jayasubba Reddy Yarava[1], Qinghui Cheng[1, 2], Tuo Wang[1*]

[1] Department of Chemistry, Michigan State University, East Lansing, MI 48824, USA

[2] State key Laboratory of Non-food Biomass Energy Technology, Institute of Biological Science and Technology, Guangxi Academy of Sciences, Nanning 530007, China

[#] These authors contributed equally

[*] Correspondence: wangtuo1@msu.edu

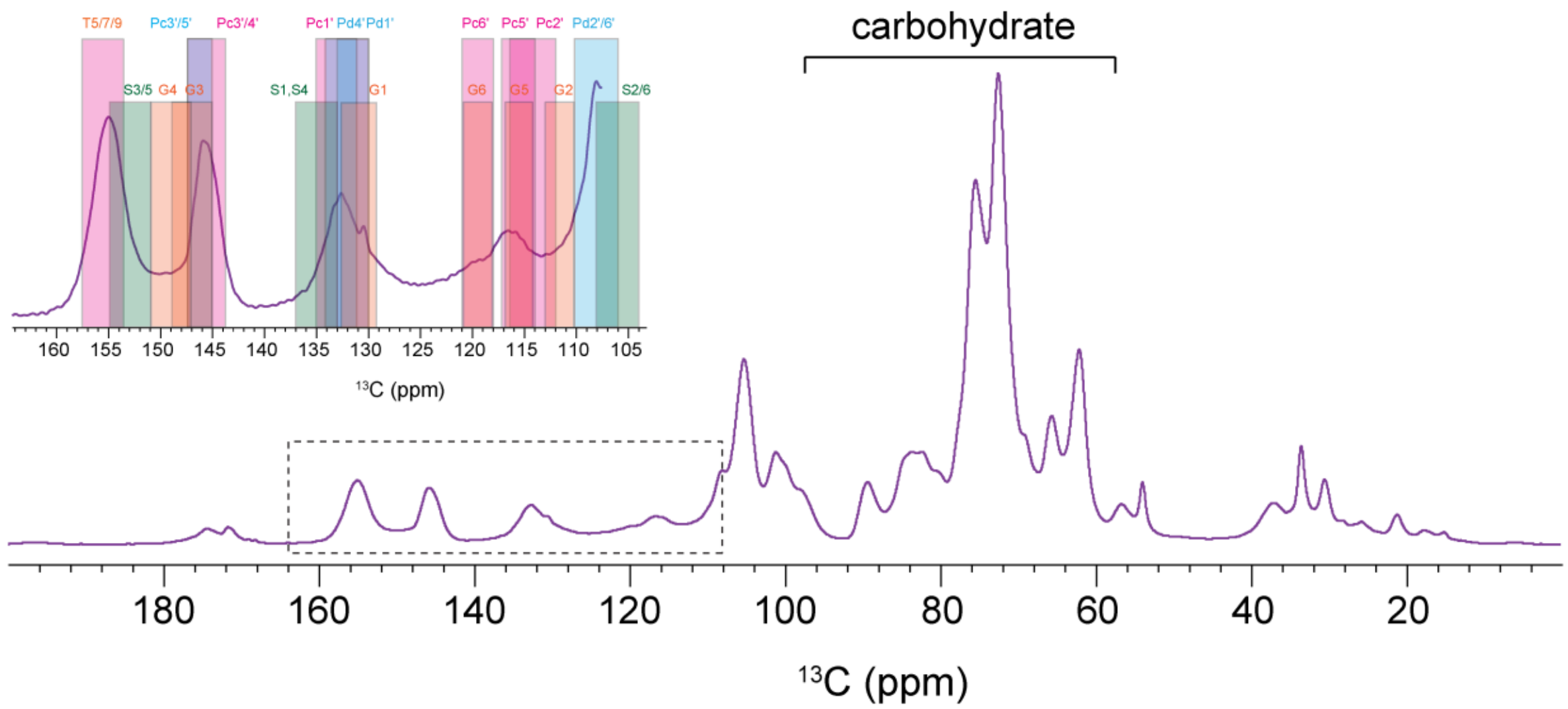

Fig. S1. Expected ^{13}C chemical shift regions for condensed tannins and lignin were mapped onto the experimental spectrum based on reported literature values. The extensive overlap between the two aromatic polymers demonstrates the limited specificity of one-dimensional ^{13}C spectra and underscores the need for multidimensional solid-state NMR approaches to resolve tannin-and lignin-associated correlations.

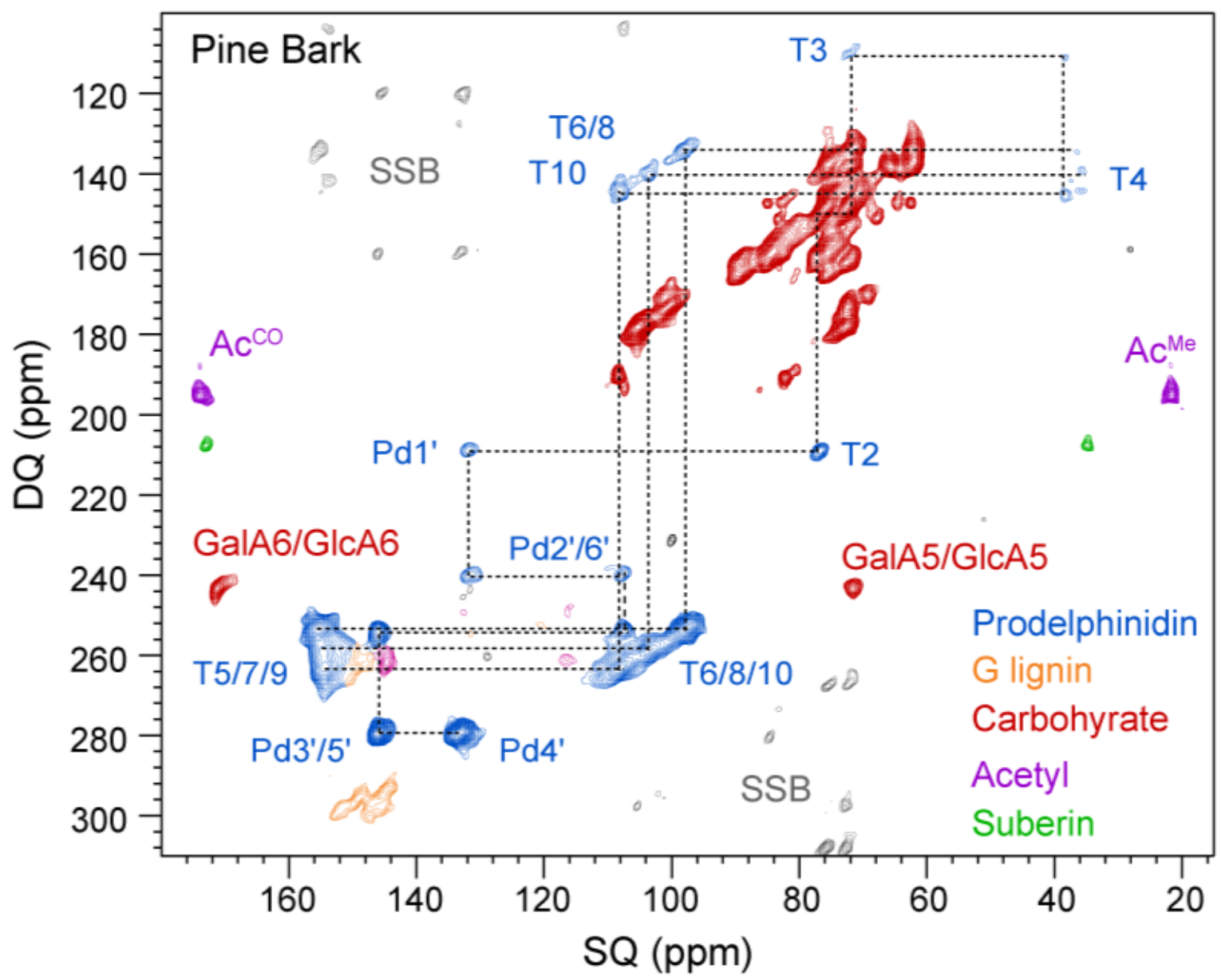

Fig. S2. Full spectral region of aromatic and carbohydrate correlations in the pine bark DQ-SQ spectrum. The DQ-SQ spectrum of pine bark is shown with assigned cell wall components color-coded as follows: carbohydrates (red), G-lignin (orange), acetyl groups (purple), suberin (green), Pd tannins (blue), and Pc tannins (magenta). Spinning sidebands (SSBs) are shown in gray. The contour features observed in the SQ 60-80 ppm and DQ 220-240 ppm region arises primarily from GalA/GlcA5 carbohydrate correlations, with additional weaker contributions from carbohydrate spinning sidebands. The full carbon-site connectivity of Pd tannins is highlighted with dashed lines. The first contour level is 5.5 standard deviations (σ), and the contour multiplication factor is 1.2.

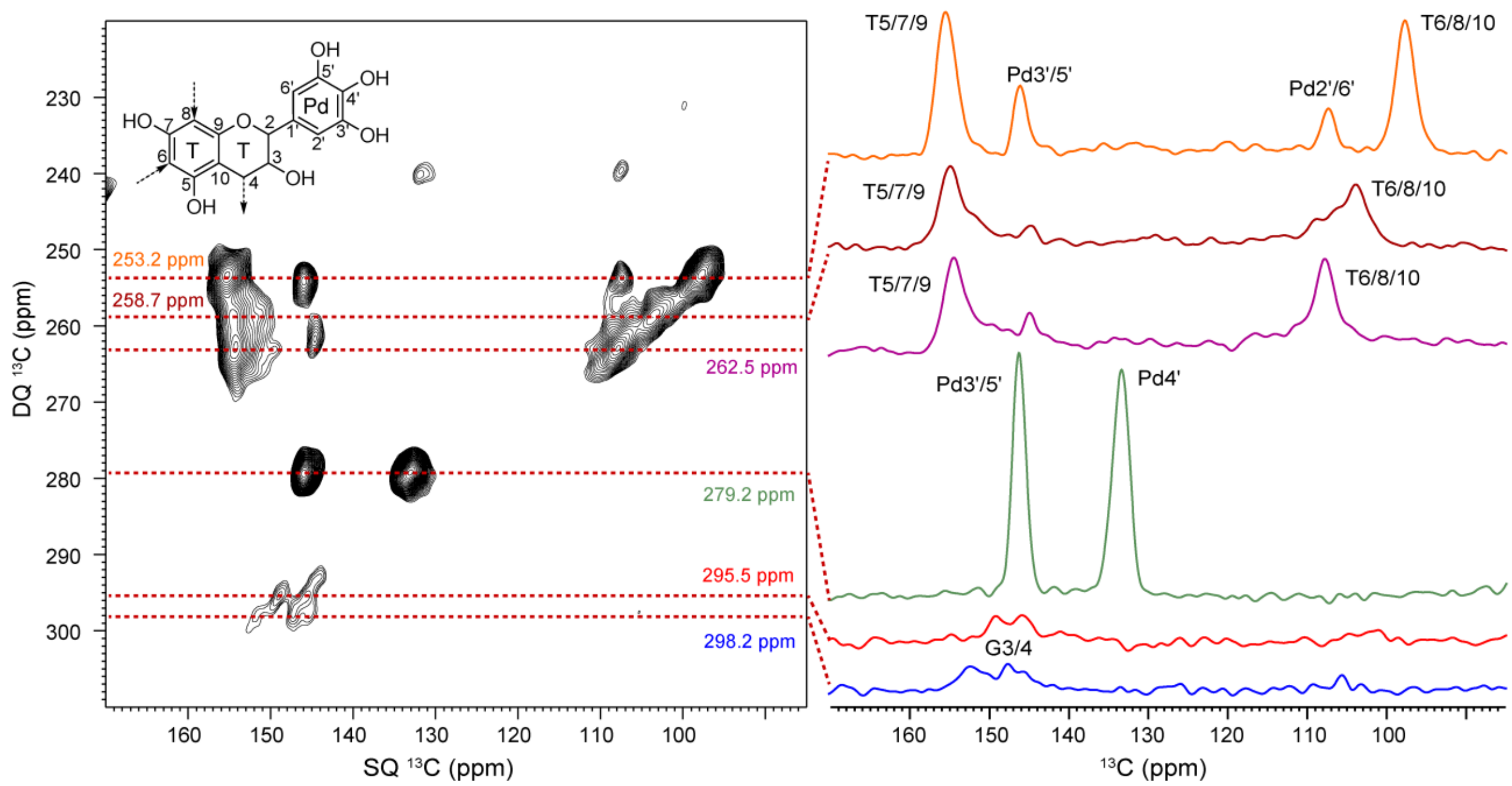

Fig. S3. Selected cross-sections of the pine bark DQ-SQ spectrum. The DQ-SQ spectrum of pine bark is shown together with selected one-dimensional SQ-dimension cross-sections extracted at representative DQ positions. The extraction positions are indicated by dashed lines, with the corresponding DQ chemical shifts labeled on the spectrum. These cross-sections illustrate the spectral resolution and relative signal intensities of key tannin and lignin correlations. The signal-to-noise ratios (SNRs) are 86-89 for the strongest peaks (Pd3′/5′ and Pd4′), 30-55 for the intermediate-intensity peaks (T5/7/9 and T6/8/10), and 7-10 for the weakest peaks (G3/4). The first contour level is 8.5 standard deviations (σ), and the contour multiplication factor is 1.2.

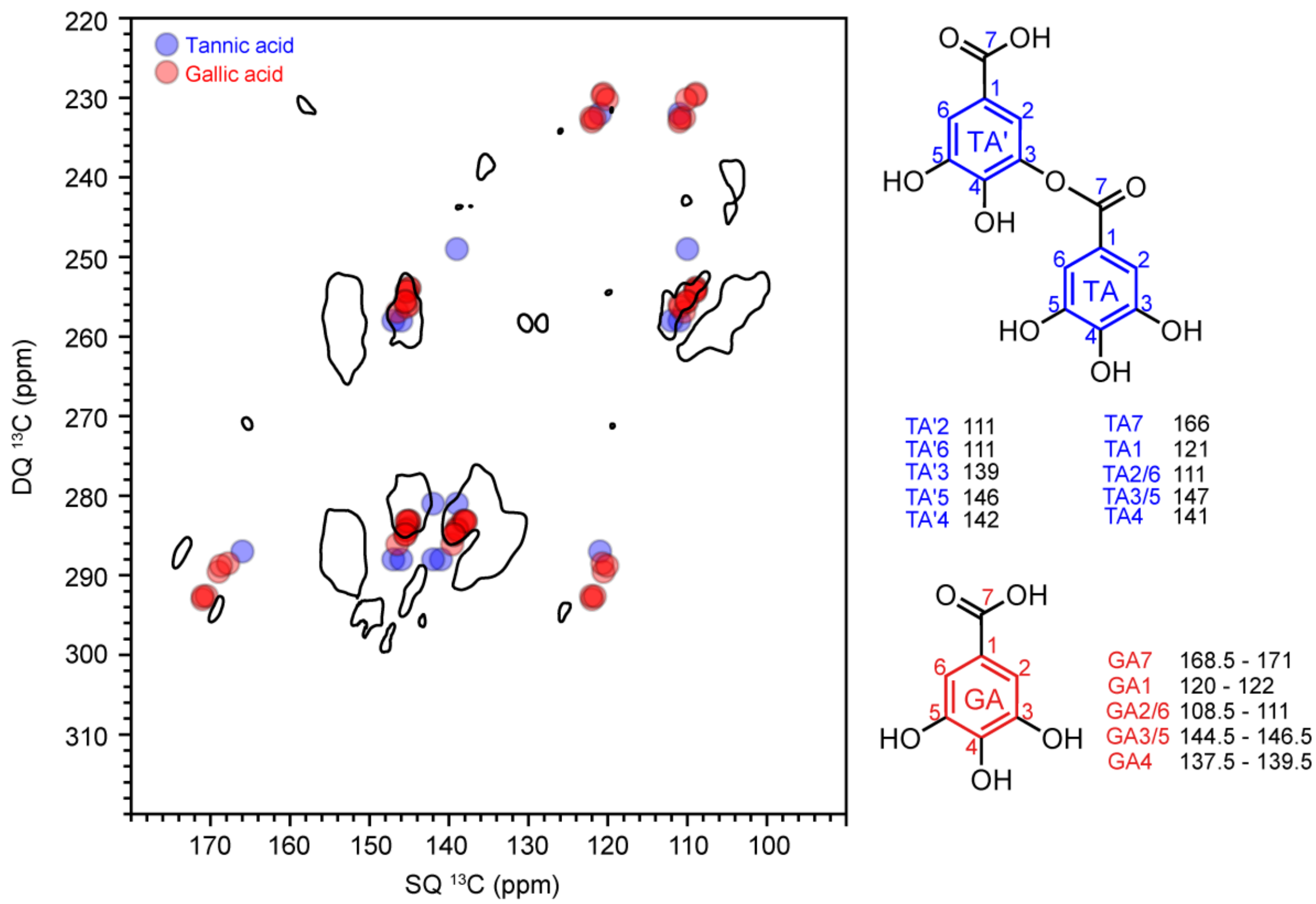

Figure S4. Mapping of potential gallic acid and tannic acid in Eucalyptus. The model structure of gallic acid (GA, red) and tannic acid (TA, blue) are shown on the right, with the chemical shift values and ranges from literature listed. The C7 (C=O) in both units have SQ chemical shifts in the range of 165-171 ppm, with DQ chemical shifts in the range of 286-294 ppm, connected to C1 at the same DQ range but SQ in the range of 120-122 ppm. Each simulated peak is 3 ppm in diameter.

Table S1. NMR experimental parameters for 2D experiments. Unavailable site is indicated as "-". τ_{cp1} and τ_{cp2}: the contact times for the first (hC) and the second (Ch and CH) CP, respectively. NS: number of scans; D1 = recycle delay between scans. TD1/2 and AQ1/2: total data points and chemical shift evolution time for dimensions 1/2, respectively. τ: half of the spin echo (INADEQUATE).

Expt.	CP (μs)		NS	D1(s)	TD1	TD2	τ (ms)	AQ1 (ms)	AQ2 (ms)	MAS (kHz)	Expt.Time	Sample
	τ_{cp1}	τ_{cp2}										
2D hCH	1200	50	8	2	384 (^{13}C)	2000 (^{1}H)	-	6.4	10.0	60	1h49m	Spruce
	1200	1200	8	2	384 (^{13}C)	2000 (^{1}H)	-	6.4	12.2	60	1h49m	
2D CP J-INADEQUATE	1000	-	64	1.6	340 (^{13}C)	1400 (^{13}C)	1.42	14.0	4.5	14	~10h	
2D CP J-INADEQUATE	1000	-	32	1.6	380 (^{13}C)	1400 (^{13}C)	1.42	14.0	5.0	14	~7h	Eucalyptus
2D CP J-INADEQUATE	2000	-	128	1.5	250 (^{13}C)	1000 (^{13}C)	1.48	2.9	10.0	18	13h30m	Pine

Table S2. ^{13}C chemical shifts of Tannin variants. Unavailable site is indicated as "-". All values are from the lignin database by Ralph and coworkers, referenced on TMS scale. Letters and numbers with an apostrophe or double apostrophe represent neighboring units.

C6′	*C5′*	*C4′*	*C3′*	*C2′*	*C1′*	C2	C3	C4	C10	C5	C6	C7	C8	C9	Compound	Solvent	Reference
107.8	146.3	133.3	146.3	107.8	130.9	83	67.7	28.1	99.4	157.2	95.3	157.8	94.8	157.3	prodelphinidin	DMSO	
106	145.5	132.5	145.5	106	133	80	71	36	98	155.5	101	156	104	157	prodelphinidin	DMSO	ChemDraw
107	144.5	134	144.5	107	132	78	72	38	103	155.7	110	152	108	154	prodelphinidin	DMSO	Stimulations
109	147	131	147	109	130	76	73	37	108	155.5	105	153	107	151	prodelphinidin	DMSO	
109.8	145.6	138.8	145.6	109.8	-	-	-	-	-	-	-	-	-	-		Acetone	Kennedy et al.(1, 2)
121	116.1	144.6	145.8	115.2	131.5	85.7	67.7	28.1	95	157.2	95.3	157.8	94.8	157.3	procyanidin	DMSO	Chem draw sti.
118.7	115.4	145.2	145.1	114.4	131.0	75.1	74.7	33.9	100.6	157.7	96.1	157.3	95.3	157.1	procyanidin	Acetone	Kennedy et al.(1, 2)
120	117	144.5	144	114.8	131.5	82.2	72.6	37	102.6	155.2	97.9	156	103.8	156	procyanidin		Xue et al.(3)
120	113.5	146.5	146.5	115.9	131	76.2	72.6	37	105.2	156	107.6	155.2	109.5	155.2	procyanidin		Xue et al.(3)
118	116.3	144	144	116	129.9	76	73	37.3	98.8	156	104.9	156	97.9	155.2	procyanidin		Newman et al.(4)
119	114.8	144	144	112	133	80.8	71.2	36.4	97.9	155.2	105.2	157	104.9	156	procyanidin		Zhang et al.(5)
119	115.9	144	144	113	135	85.9	73	28.8	104.9	156	109.5	157	107.6	155.2	procyanidin		Zeller et al.(6)
119	116	146	146	115	132	78	73	37	103	154	97	154	96	154	procyanidin		Zheng et al.(7)
119	116	146	146	115	132	79	72	37	103	155	105	155	107	154	procyanidin		Zheng et al.(7)
-	-	-	-	-	-	-		104.9	-	-	107	-	105	-	C4-C6/C4-C8		Newman et al.(4) Zhang et al.(5)

Table S3. ^{13}C chemical shifts of S lignin variants. Unavailable site is indicated as "-". All values are from the lignin database by Ralph and coworkers(8), referenced on TMS scale. Letters and numbers with an apostrophe or double apostrophe represent neighboring units.

C1	C2	C3	C4	C5	C6	α	β	γ	Unit Type	Linkage Type	Polymer Note	Compound#	Solvent
-	105.64	152.69	136.18	152.69	105.64	-	-	-	S:4-O-β'	4-O-β'	G-b-**S** (S1: C-H)	179	DMSO
-	105.65	153.29	135.65	153.29	105.65	-	-	-	S:4-O-β'	4-O-β'	G-b-**S** (S1: C-H)	217	DMSO
-	105.46	152.43	135.80	152.43	105.46	-	-	-	S:4-O-β'	4-O-β'	S-b-**S** (S1: C-H)	21	DMSO
131.90	105.20	152.47	134.12	152.47	105.20	65.58	-	-	S:4-O-β'	4-O-β'	S-b-**S** (α)(α: CH2-OAc)	229	DMSO
133.80	106.28	152.27	132.90	152.27	106.28	21.31	-	-	S:4-O-β'	4-O-β'	G-b-**S** (α)(α: -CH3)	242	DMSO
132.88	106.34	152.34	133.92	152.34	106.34	21.31	-	-	S:4-O-β'	4-O-β'	G-b-**S** (α)(α: -CH3)	243	DMSO
137.62	105.58	152.76	133.46	152.76	105.58	37.64	24.10	13.73	S:4-O-β'	4-O-β'	G-b-**S** (αβγ)(α,β: -CH2)(γ: -CH3)	3026	DMSO
137.61	105.67	152.79	133.46	152.79	105.67	37.60	24.18	13.82	S:4-O-β'	4-O-β'	S-b-**S** (αβγ)(α,β: -CH2)(γ: -CH3)	3028	DMSO
134.77	103.29	152.61	136.89	152.61	103.29	85.12	53.61	71.27	S:4-O-β'	4-O-β', β-β"	G-b-**S**-r-S (αβγ)(α-O-γ')(β-β')(γ-O-α')	183	DMSO
134.42	103.28	152.55	136.79	152.55	103.28	85.10	53.59	71.26	S:4-O-β'	4-O-β', β-β"	S-b-**S**-r-S (αβγ)(α-O-γ')(β-β')(γ-O-α')	198	DMSO
134.79	103.24	152.60	136.59	152.60	103.24	86.93	52.93	62.71	S:4-O-β'	4-O-β', α-O-4', β-5'	G-b-**S**-c-GA (αβγ)(α-O-4')(β-5')(γ-OH)	181	DMSO
133.62	106.95	153.61	141.68	153.61	106.95	196.70	26.55	-	S:4-O-β'	4-O-β'	S-b-**S** (αβ)(α: C=O)(β: -CH3)	53	DMSO
133.69	105.85	151.71	140.22	151.71	105.85	195.71	26.54	-	S:4-O-β'	4-O-β'	S-b-**S** (αβ)(α: C=O)(β: -CH3)	79	DMSO
131.79	105.44	151.62	132.05	151.62	105.44	196.51	83.67	62.39	S:4-OAc	β-O-4'	**S**-b-S (αβγ)(α: C=O)(γ-OH)(β-O-4')	79	DMSO
130.19	105.42	151.98	132.55	151.98	105.42	196.08	80.64	62.41	S:4-OAc	β-O-4'	**S**-b-H (αβγ)(α: C=O)(γ-OH)(β-O-4')	80	DMSO
130.46	105.45	151.87	132.46	151.87	105.45	196.14	81.51	62.32	S:4-OAc	β-O-4'	**S**-b-G (αβγ)(α: C=O)(γ-OH)(β-O-4')	81	DMSO
131.97	104.14	147.25	134.50	147.25	104.14	71.54	87.11	60.15	S:4-OH	β-O-4'	**S**-b-S (αβγ)(α,γ: -OH)(β-O-4')	89	DMSO
132.13	104.30	147.37	134.32	147.37	104.30	71.59	87.18	60.18	S:4-OH	β-O-4'	**S**-b-S (αβγ)(α,γ: -OH)(β-O-4')	243	DMSO
131.89	104.07	147.31	134.26	147.31	104.07	70.88	84.03	60.01	S:4-OH	β-O-4'	**S**-b-G (αβγ)(α,γ: -OH)(β-O-4')	90	DMSO
131.93	104.06	147.30	134.24	147.30	104.06	70.87	84.39	59.99	S:4-OH	β-O-4'	**S**-b-**G** (αβγ)(α,γ: -OH)(β-O-4')	88	DMSO
130.21	104.15	147.39	134.33	147.39	104.15	70.90	84.58	60.05	S:4-OH	β-O-4'	**S**-b-G (αβγ)(α,γ: -OH)(β-O-4')	245	DMSO
131.93	104.13	147.34	134.31	147.34	104.13	70.88	84.44	60.00	S:4-OH	β-O-4'	**S**-b-**G** (αβγ)(α,γ: -OH)(β-O-4')	212	DMSO
131.36	104.29	147.40	134.86	147.40	104.29	72.38	86.20	59.90	S:4-OH	β-O-4'	**S**-b-S-r-S (αβγ)(α,γ: -OH)(β-O-4')	198	DMSO
128.54	106.89	147.65	133.70	147.65	106.89	37.35	83.32	61.88	S:4-OH	β-O-4'	**S**-b-S (αβγ)(α: -CH2)(γ-OH)(β-O-4')	3028	DMSO
128.24	106.83	147.64	133.78	147.64	106.83	36.90	80.56	61.84	S:4-OH	β-O-4'	**S**-b-G (αβγ)(α: -CH2)(γ-OH)(β-O-4')	3027	DMSO
132.42	103.65	147.88	134.86	147.88	103.65	85.30	53.73	71.10	S:4-OH	β-β'	S-b-S-r-**S** (αβγ)(α-O-γ')(β-β')(γ-O-α')	198	DMSO
131.38	103.68	147.90	134.89	147.90	103.68	85.32	53.76	71.13	S:4-OH	β-β'	G-b-**S**-r-S (αβγ)(α-O-γ')(β-β')(γ-O-α')	183	DMSO
131.36	103.59	147.82	134.81	147.82	103.59	85.26	53.59	70.99	S:4-OH	β-β'	**S**-r-**S** (αβγ)(α-O-γ')(β-β')(γ-O-α')	117	DMSO
126.22	106.56	147.30	140.92	147.30	106.56	194.71	82.80	61.95	S:4-OH	β-O-4'	**S**-b-S (αβγ)(α: C=O)(γ-OH)(β-O-4')	21	DMSO
-	106.53	154.19	137.16	154.19	106.53	-	-	-	S:4-O-β'	4-O-β'	G-b-**S** (S1: C-H)	179	Acetone
-	106.49	154.61	137.09	154.61	106.49	-	-	-	S:4-O-β'	4-O-β'	G-b-**S** (S1: C-H)	217	Acetone
-	106.32	153.86	137.37	153.86	106.32	-	-	-	S:4-O-β'	4-O-β'	S-b-**S** (S1: C-H)	21	Acetone
-	106.22	153.77	137.01	153.77	106.22	-	-	-	S:4-O-β'	4-O-β'	S-b-**S** (S1: C-H)	2082	Acetone
133.40	106.29	154.13	136.29	154.13	106.29	66.71	-	-	S:4-O-β'	4-O-β'	S-b-**S** (α)(α: CH2-OAc)	229	Acetone
134.82	107.09	153.60	134.65	153.60	107.09	21.77	-	-	S:4-O-β'	4-O-β'	G-b-**S** (α)(α: -CH3)	242	Acetone
134.55	107.05	153.52	134.78	153.52	107.05	21.78	-	-	S:4-O-β'	4-O-β'	G-b-**S** (α)(α: -CH3)	243	Acetone
139.25	106.55	154.09	134.92	154.09	106.55	38.91	25.30	14.06	S:4-O-β'	4-O-β'	G-b-**S** (αβγ)(α,β: -CH2)(γ: -CH3)	3026	Acetone
139.10	106.57	154.07	134.94	154.07	106.57	38.80	25.30	14.06	S:4-O-β'	4-O-β'	S-b-**S** (αβγ)(α,β: -CH2)(γ: -CH3)	3028	Acetone
135.67	104.09	154.15	139.05	154.15	104.09	86.57	55.23	72.57	S:4-O-β'	4-O-β', β-β"	G-b-**S**-r-S (αβγ)(α-O-γ')(β-β')(γ-O-α')	183	Acetone
135.86	104.10	154.16	139.07	154.16	104.10	86.61	55.27	72.61	S:4-O-β'	4-O-β', β-β"	S-b-**S**-r-S (αβγ)(α-O-γ')(β-β')(γ-O-α')	198	Acetone
133.80	104.12	154.20	139.10	154.20	104.12	86.57	55.46	72.54	S:4-O-β'	4-O-β', β-β"	G-b-**S**-r-G (αβγ)(α-O-γ')(β-β')(γ-O-α')	3064	Acetone
136.10	104.10	154.26	139.09	154.26	104.10	88.36	54.91	64.62	S:4-O-β'	4-O-β', α-O-4', β-5'	G-b-**S**-c-GA (αβγ)(α-O-4')(β-5')(γ-OH)	181	Acetone
131.09	106.94	154.57	139.26	154.57	106.94	153.45	129.11	193.90	S:4-O-β'	4-O-β'	G-b-**S** (αβγ)(α=β)(γ: C=O)	3071	Acetone
133.66	106.52	153.21	133.80	153.21	106.52	196.65	86.08	63.67	S:4-OAc	β-O-4'	**S**-b-S (αβγ)(α: C=O)(γ-OH)(β-O-4')	79	Acetone

131.80	106.40	153.47	134.34	153.47	106.40	196.21	82.26	63.94	S:4-OAc	β-O-4'	**S**-b-H (αβγ)(α: C=O)(γ-OH)(β-O-4')	80	Acetone
132.37	106.56	153.35	134.22	153.35	106.56	196.27	83.76	63.91	S:4-OAc	β-O-4'	**S**-b-G (αβγ)(α: C=O)(γ-OH)(β-O-4')	81	Acetone
135.01	106.51	153.06	133.59	153.06	106.51	196.25	86.41	63.35	S:4-OAc	β-O-4'	**S**-b-S (αβγ)(α: C=O)(γ-OH)(β-O-4')	2082	Acetone
133.62	106.95	153.61	141.68	153.61	106.95	196.70	26.55	-	S:4-O-β'	4-O-β'	S-b-**S** (αβ)(α: C=O)(β: -CH3)	53	Acetone
134.87	106.62	153.21	141.29	153.21	106.62	195.71	26.54	-	S:4-O-β'	4-O-β'	S-b-**S** (αβ)(α: C=O)(β: -CH3)	79	Acetone
132.60	105.46	148.30	136.11	148.30	105.46	74.22	89.69	61.41	S:4-OH	β-O-4'	**S**-b-S (αβγ)(α,γ: -OH)(β-O-4')	89	Acetone
132.54	105.39	148.23	136.00	148.23	105.39	74.29	89.56	61.31	S:4-OH	β-O-4'	**S**-b-S (αβγ)(α,γ: -OH)(β-O-4')	243	Acetone
132.72	105.28	148.34	136.07	148.34	105.28	73.97	88.19	61.89	S:4-OH	β-O-4'	**S**-b-G (αβγ)(α,γ: -OH)(β-O-4')	90	Acetone
132.80	105.36	148.38	136.12	148.38	105.36	74.05	88.50	61.94	S:4-OH	β-O-4'	**S**-b-**G** (αβγ)(α,γ: -OH)(β-O-4')	88	Acetone
132.84	105.41	148.40	136.15	148.40	105.41	74.05	88.73	61.89	S:4-OH	β-O-4'	**S**-b-G (αβγ)(α,γ: -OH)(β-O-4')	245	Acetone
132.82	105.41	148.50	136.17	148.50	105.41	74.06	88.56	61.95	S:4-OH	β-O-4'	**S**-b-**G** (αβγ)(α,γ: -OH)(β-O-4')	212	Acetone
132.73	104.91	148.38	135.86	148.38	104.91	73.63	87.87	61.03	S:4-OH	β-O-4'	**S**-b-S-r-S (αβγ)(α,γ: -OH)(β-O-4')	198	Acetone
129.69	107.83	148.44	135.24	148.44	107.83	38.38	85.20	62.93	S:4-OH	β-O-4'	**S**-b-S (αβγ)(α: -CH2)(γ-OH)(β-O-4')	3028	Acetone
129.59	107.93	148.48	135.34	148.48	107.93	38.21	83.47	63.46	S:4-OH	β-O-4'	**S**-b-G (αβγ)(α: -CH2)(γ-OH)(β-O-4')	3027	Acetone
133.13	104.51	148.68	136.23	148.68	104.51	86.73	55.42	72.40	S:4-OH	β-β'	S-b-S-r-**S** (αβγ)(α-O-γ')(β-β')(γ-O-α')	198	Acetone
133.10	104.50	148.67	136.20	148.67	104.50	86.72	55.39	72.38	S:4-OH	β-β'	G-b-**S**-r-S (αβγ)(α-O-γ')(β-β')(γ-O-α')	183	Acetone
133.24	104.52	148.69	136.23	148.69	104.52	86.81	55.33	72.37	S:4-OH	β-β'	**S**-r-**S** (αβγ)(α-O-γ')(β-β')(γ-O-α')	117	Acetone
133.30	104.55	148.74	136.68	148.74	104.55	88.74	54.78	64.52	S:4-OH	α-O-4' + β-5'	**S**-c-G (αβγ)(α-O-4')(β-5')(γ-OH)	3063	Acetone
127.86	107.69	148.31	141.99	148.31	107.69	195.15	86.21	63.66	S:4-OH	β-O-4'	**S**-b-S (αβγ)(α: C=O)(γ-OH)(β-O-4')	21	Acetone

Table S4. ^{13}C chemical shifts of G lignin 4-OH moiety. Unavailable site is indicated as "-". All values are from the lignin database by Ralph and coworkers(8), referenced on TMS scale. Letters with an apostrophe represent neighboring units.

C1	C2	C3	C4	C5	C6	α	β	γ	Unit Type	Linkage Type	Polymer Note	Compound#	Solvent
132.26	110.43	147.53	145.91	115.15	118.64	85.17	53.59	70.91	G:4-OH	β-β'	G-r-G (αβγ)(α-O-γ')(β-β')(γ-O-α')	2020	DMSO
128.41	110.96	147.35	146.24	115.28	119.48	83.07	51.54	174.99	G:4-OH	β-β'	G-r-G (αβγ)(α-O-γ')(β-β')(γ: C=O)	2027	DMSO
130.80	110.88	147.96	147.03	115.80	120.06	85.45	50.42	71.06	G:4-OH	β-β'	G-r-G (αβγ)(α-O-γ')(β-β')(γ-O-α')	2027	DMSO
134.17	110.60	148.32	146.86	115.52	119.59	86.62	55.23	72.20	G:4-OH	β-β'	G-r-G (αβγ)(α-O-γ')(β-β')(γ-O-α')	2020	Acetone
129.78	111.21	147.99	147.15	115.39	120.32	84.43	52.43	174.93	G:4-OH	β-β'	G-r-G (αβγ)(α-O-γ')(β-β')(γ: C=O)	2027	Acetone
132.60	110.68	148.62	147.82	115.80	120.06	86.32	52.02	72.08	G:4-OH	β-β'	G-r-G (αβγ)(α-O-γ')(β-β')(γ-O-α')	2027	Acetone
133.48	110.24	148.39	147.10	115.65	119.17	84.56	55.35	177.77	G:4-OH	β-β'	G-r-G (αβγ)(α-O-γ')(β-β')(γ: C=-O)	2028	Acetone
129.22	109.50	148.54	147.24	115.87	118.57	81.09	46.14	69.98	G:4-OH	β-β'	G-r-G (αβγ)(α-O-γ')(β-β')(γ-O-α')	2028	Acetone
130.82	110.56	147.59	144.93	115.20	119.25	92.37	44.71	17.17	G:4-OH	α-O-4' + β-5'	G-c-G (αβγ)(α-O-4')(β-5')(γ: -CH3)	66	DMSO
130.93	109.74	147.59	146.07	113.29	119.25	92.55	44.51	17.25	G:4-OH	α-O-4' + β-5'	G-c-G (αβγ)(α-O-4')(β-5')(γ: -CH3)	67	DMSO
132.39	110.37	147.13	146.42	115.00	118.58	87.26	53.02	62.98	G:4-OH	α-O-4' + β-5'	G-c-GA (αβγ)(α-O-4')(β-5')(γ: -OH)	2004	DMSO
133.03	110.69	148.39	146.59	115.55	120.22	93.80	46.43	17.83	G:4-OH	α-O-4' + β-5'	G-c-G (αβγ)(α-O-4')(β-5')(γ: -CH3)	66	Acetone
132.81	110.66	148.36	147.53	114.30	120.21	93.96	46.21	17.86	G:4-OH	α-O-4' + β-5'	G-c-G (αβγ)(α-O-4')(β-5')(γ: -CH3)	67	Acetone
134.36	110.48	148.36	147.27	115.67	119.57	88.51	54.73	64.60	G:4-OH	α-O-4' + β-5'	G-c-GA (αβγ)(α-O-4')(β-5')(γ: -OH)	2004	Acetone
133.75	110.59	148.46	147.55	115.76	119.73	89.39	54.25	64.32	G:4-OH	α-O-4' + β-5'	G-c-GA (αβγ)(α-O-4')(β-5')(γ: -OH)	2021	Acetone
133.42	110.65	148.49	147.67	115.79	119.82	89.87	53.84	64.22	G:4-OH	α-O-4' + β-5'	G-c-G (αβγ)(α-O-4')(β-5')(γ: -OH)	3061	Acetone
128.24	111.91	147.11	146.07	114.74	120.34	79.01	82.53	59.74	G:4-OH	α-O-4' + β-O-4''	GA-a-G-b-GA (αβγ)(α-O-4')(β-O-4'')(γ: -OH)	2015	DMSO
129.59	111.82	147.18	146.31	115.14	120.11	83.34	85.57	60.66	G:4-OH	α-O-4' + β-O-4''	DiBenzoDiOxicin	277	DMSO
129.10	111.70	148.30	147.80	115.70	121.40	76.70	79.30	61.90	G:4-OH	α-O-5' + β-O-4'	G-b'-GA (αβγ)(α-O-5')(β-O-4')(γ: -OH)	3068	Acetone
128.90	112.00	148.10	148.50	115.80	121.60	77.00	79.80	61.60	G:4-OH	α-O-5' + β-O-4'	G-b'-G (αβγ)(α-O-5')(β-O-4')(γ: -OH)	3069	Acetone
130.39	112.11	148.10	147.18	115.23	121.49	81.15	85.31	61.75	G:4-OH	α-O-4' + β-O-4''	GA-a-G-b-GA (αβγ)(α-O-4')(β-O-4'')(γ: -OH)	2015	Acetone
128.80	111.70	150.10	147.80	115.70	121.40	76.90	79.50	61.60	G:4-OH	α-O-5' + β-O-4'	G-b'-G (αβγ)(α-O-5')(β-O-4')(γ: -OH)	3070	Acetone
131.38	111.94	148.16	147.43	115.56	121.33	85.18	87.71	62.97	G:4-OH	α-O-4' + β-O-4''	DiBenzoDiOxicin	277	Acetone
130.25	112.08	148.23	147.73	115.64	121.57	85.11	87.48	62.29	G:4-OH	α-O-4' + β-O-4''	DiBenzoDiOxicin	3062	Acetone
133.15	111.35	146.86	145.35	114.48	119.43	71.60	83.67	60.04	G:4-OH	β-O-4'	G-b-G (αβγ)(α,γ: -OH)(β-O-4')	101e	DMSO
132.86	110.96	146.89	145.33	114.57	118.92	70.90	84.37	60.02	G:4-OH	β-O-4'	G-b-G (αβγ)(α,γ: -OH)(β-O-4')	102t	DMSO
132.79	111.01	146.83	145.29	114.62	119.22	71.53	87.29	60.11	G:4-OH	β-O-4'	G-b-S (αβγ)(α,γ: -OH)(β-O-4')	242	DMSO
132.82	110.97	146.77	145.23	114.56	119.14	71.48	87.20	60.09	G:4-OH	β-O-4'	G-b-S (αβγ)(α,γ: -OH)(β-O-4')	134	DMSO
132.86	110.93	146.76	145.20	114.55	119.10	71.40	86.96	60.08	G:4-OH	β-O-4'	G-b-S (αβγ)(α,γ: -OH)(β-O-4')	179	DMSO
132.89	110.98	146.87	145.31	114.57	118.93	70.91	84.74	60.02	G:4-OH	β-O-4'	G-b-G (αβγ)(α,γ: -OH)(β-O-4')	131	DMSO
133.15	110.81	146.86	145.18	114.56	119.19	71.98	86.14	59.64	G:4-OH	β-O-4'	G-b-S-c-GA (αβγ)(α,γ: -OH)(β-O-4')	181	DMSO
133.26	110.96	145.32	146.96	114.65	119.36	72.12	86.14	59.84	G:4-OH	β-O-4'	G-b-S-r-S (αβγ)(α,γ: -OH)(β-O-4')	183	DMSO
130.36	111.05	147.00	146.13	114.69	119.04	70.96	84.95	60.10	G:4-OH	β-O-4'	G-b-G (αβγ)(α,γ: -OH)(β-O-4')	248t	DMSO
133.23	111.47	147.00	147.00	114.60	119.54	71.67	83.75	60.16	G:4-OH	β-O-4'	G-b-GA (αβγ)(α,γ: -OH)(β-O-4')	2014e	DMSO
132.97	111.05	147.03	145.46	114.71	119.05	73.83	88.37	61.88	G:4-OH	β-O-4'	G-b-GA (αβγ)(α,γ: -OH)(β-O-4')	2013t	DMSO
129.32	113.58	147.15	144.64	115.11	121.68	36.86	83.46	61.69	G:4-OH	β-O-4'	G-b-S (αβγ)(α: -CH2)(γ: -OH)(β-O-4')	217	DMSO
129.24	113.55	147.03	144.55	115.01	121.57	36.77	83.40	61.58	G:4-OH	β-O-4'	G-b-S (αβγ)(α: -CH2)(γ: -OH)(β-O-4')	135	DMSO
129.36	113.58	147.11	144.60	115.06	121.64	36.86	83.39	61.65	G:4-OH	β-O-4'	G-b-S (αβγ)(α: -CH2)(γ: -OH)(β-O-4')	3026	DMSO
129.16	113.64	147.26	144.79	115.19	121.63	36.52	80.83	61.82	G:4-OH	β-O-4'	G-b-G (αβγ)(α: -CH2)(γ: -OH)(β-O-4')	3025	DMSO
134.23	111.38	147.93	146.61	115.12	120.45	73.82	86.72	61.81	G:4-OH	β-O-4'	G-b-G (αβγ)(α,γ: -OH)(β-O-4')	101e	Acetone
133.81	111.41	148.00	146.82	115.21	120.57	73.93	88.58	61.90	G:4-OH	β-O-4'	G-b-G (αβγ)(α,γ: -OH)(β-O-4')	102t	Acetone

133.60	111.45	147.90	146.75	115.22	120.73	74.08	89.83	61.19	G:4-OH	β-O-4'	G-b-S (αβγ)(α,γ: -OH)(β-O-4')	242	Acetone
133.67	111.51	147.95	146.79	115.24	120.75	74.10	89.82	61.35	G:4-OH	β-O-4'	G-b-S (αβγ)(α,γ: -OH)(β-O-4')	134	Acetone
133.71	111.51	147.95	146.81	115.23	120.76	74.07	89.76	61.31	G:4-OH	β-O-4'	G-b-S (αβγ)(α,γ: -OH)(β-O-4')	179	Acetone
133.85	111.45	148.04	146.83	115.24	120.59	73.95	88.72	61.89	G:4-OH	β-O-4'	G-b-G (αβγ)(α,γ: -OH)(β-O-4')	131	Acetone
133.73	110.89	147.97	146.44	115.20	120.01	73.37	87.87	60.97	G:4-OH	β-O-4'	G-b-S-c-GA (αβγ)(α,γ: -OH)(β-O-4')	181	Acetone
133.74	110.93	146.43	147.96	115.21	120.04	73.38	87.78	60.97	G:4-OH	β-O-4'	G-b-S-r-S (αβγ)(α,γ: -OH)(β-O-4')	183	Acetone
133.16	111.40	148.02	147.82	115.23	120.57	73.95	88.90	61.82	G:4-OH	β-O-4'	G-b-G (αβγ)(α,γ: -OH)(β-O-4')	248t	Acetone
134.24	111.44	147.96	146.65	115.13	120.46	73.81	86.63	61.82	G:4-OH	β-O-4'	G-b-GA (αβγ)(α,γ: -OH)(β-O-4')	2014e	Acetone
133.82	111.42	148.03	146.83	115.21	120.54	73.83	88.37	61.88	G:4-OH	β-O-4'	G-b-GA (αβγ)(α,γ: -OH)(β-O-4')	2013t	Acetone
134.10	110.92	146.47	147.98	115.20	120.04	73.38	87.86	60.98	G:4-OH	β-O-4'	G-b-S-r-G (αβγ)(α,γ: -OH)(β-O-4')	3064	Acetone
130.76	113.89	148.05	145.71	115.52	122.81	37.97	85.29	62.78	G:4-OH	β-O-4'	G-b-S (αβγ)(α: -CH2)(γ: -OH)(β-O-4')	217	Acetone
130.80	113.93	148.04	145.80	115.51	122.80	38.03	85.30	62.84	G:4-OH	β-O-4'	G-b-S (αβγ)(α: -CH2)(γ: -OH)(β-O-4')	135	Acetone
130.81	113.87	148.03	145.78	115.50	122.78	38.00	85.24	62.77	G:4-OH	β-O-4'	G-b-S (αβγ)(α: -CH2)(γ: -OH)(β-O-4')	3026	Acetone
130.58	113.99	148.05	145.88	115.49	122.83	37.74	83.67	63.38	G:4-OH	β-O-4'	G-b-G (αβγ)(α: -CH2)(γ: -OH)(β-O-4')	3025	Acetone
-	111.71	147.49	152.22	114.48	123.51	194.69	81.32	62.52	G:4-OH	β-O-4'	G-b-G (αβγ)(α: C=O)(γ: -OH)(β-O-4')	106	DMSO
128.72	112.48	148.36	152.76	115.50	124.66	195.64	83.69	64.11	G:4-OH	β-O-4'	G-b-G (αβγ)(α: C=O)(γ: -OH)(β-O-4')	106	Acetone

Table S5. ^{13}C chemical shifts of G lignin in type-A linkage. Unavailable site is indicated as “-”. All values are from the lignin database by Ralph and coworkers(8), referenced on TMS scale. Letters with an apostrophe or double apostrophe represent neighboring units.

C1	C2	C3	C4	C5	C6	α	β	γ	Unit Type	Linkage Type	Polymer Note	Compound #	Solvent
133.01	113.43	149.57	145.44	116.28	120.84	20.63	-	-	G:4-O-β'	4-O-β'	G-b-G (α)(α: -CH3)	248t	DMSO
132.00	113.34	149.46	145.98	115.95	120.76	20.57	-	-	G:4-O-β'	4-O-β'	S-b-G (α)(α:-CH3)	245	DMSO
135.45	110.97	149.39	146.93	115.43	118.58	62.74	-	-	G:4-O-β'	4-O-β'	S-b-G (α)(α-OH)	212	DMSO
-	109.65	149.23	146.73	115.29	117.20	67.80	25.84	-	G:4-O-β'	4-O-β'	S-b-G (αβ)(α-OH)(β:-CH3)	88	DMSO
-	109.79	149.31	146.87	115.62	117.24	67.78	25.80	-	G:4-O-β'	4-O-β'	G-b-G (αβ)(α-OH)(β: -CH3)	131	DMSO
135.30	112.89	149.69	145.45	115.70	120.21	36.97	24.29	13.75	G:4-O-β'	4-O-β'	G-b-G (αβγ)(α,β: -CH2)(γ: -CH3)	3025	DMSO
135.09	112.74	149.53	145.41	115.44	120.11	36.90	24.22	13.67	G:4-O-β'	4-O-β'	S-b-G (αβγ)(α,β: -CH2)(γ: -CH3)	3027	DMSO
128.82	109.93	149.80	147.36	116.15	119.00	130.49	128.47	61.52	G:4-O-β'	4-O-β'	GA-a-G-b-GA (αβγ)(α=β)(γ: -OH)	2015	DMSO
130.18	109.84	149.70	147.88	115.53	119.05	128.60	128.58	61.65	G:4-O-β'	4-O-β'	G-b-GA (αβγ)(α=β)(γ: -OH)	2013t	DMSO
130.08	109.93	149.75	147.60	115.60	119.05	128.60	128.52	61.65	G:4-O-β'	4-O-β'	G-b-GA (αβγ)(α=β)(γ: -OH)	2014e	DMSO
-	112.67	149.13	146.94	114.98	120.48	-	-	-	G:4-O-β'	4-O-β'	G-b-G (G1: C-H))	106	DMSO
-	112.68	149.14	146.88	114.58	120.48	-	-	-	G:4-O-β'	4-O-β'	G-b-G (G1: C-H))	110	DMSO
-	112.50	149.62	148.32	115.79	120.60	-	-	-	G:4-O-β'	4-O-β'	G-b-G (G1: C-H)	102t	DMSO
-	112.60	149.68	148.00	115.86	120.56	-	-	-	G:4-O-β'	4-O-β'	G-b-G (G1: C-H)	101e	DMSO
-	112.50	149.62	148.32	115.79	120.60	-	-	-	G:4-O-β'	4-O-β'	G-b-G (G1: C-H)	102t	DMSO
-	112.38	149.55	148.18	115.52	120.58	-	-	-	G:4-O-β'	4-O-β'	S-b-G (G1: C-H)	90	DMSO
132.75	111.05	148.67	150.96	113.02	122.65	195.02	26.23	-	G:4-O-β'	4-O-β'	S-b-G (αβ)(α: C=O)(β: -CH3)	81	DMSO
130.24	110.84	148.52	151.61	112.24	122.56	196.19	26.25		G:4-O-β'	4-O-β'	G-b-G (αβ)(α: C=O)(β: -CH3)	52	DMSO
133.82	114.17	151.47	146.80	120.23	122.19	21.06	-	-	G:4-O-β'	4-O-β'	G-b-G (α)(α: -CH3)	248t	Acetone
133.10	114.20	151.50	147.36	120.10	122.19	21.06	-	-	G:4-O-β'	4-O-β'	S-b-G (α)(α:-CH3)	245	Acetone
137.97	111.95	151.54	148.42	119.60	120.02	64.54	-	-	G:4-O-β'	4-O-β'	S-b-G (α)(α-OH)	212	Acetone
-	110.58	151.38	148.27	118.65	119.45	69.73	26.23	-	G:4-O-β'	4-O-β'	S-b-G (αβ)(α-OH)(β:-CH3)	88	Acetone
-	110.67	151.45	148.31	118.70	119.68	69.74	26.21	-	G:4-O-β'	4-O-β'	G-b-G (αβ)(α-OH)(β: -CH3)	131	Acetone
-	113.85	151.63	146.82	118.72	121.37	38.22	25.39	14.01	G:4-O-β'	4-O-β'	G-b-G (αβγ)(α,β: -CH2)(γ: -CH3)	3025	Acetone
-	113.81	151.57	146.85	118.54	121.36	38.23	25.40	14.01	G:4-O-β'	4-O-β'	S-b-G (αβγ)(α,β: -CH2)(γ: -CH3)	3027	Acetone
132.74	110.93	151.71	148.61	119.04	120.15	129.85	129.45	63.25	G:4-O-β'	4-O-β'	GA-a-G-b-GA (αβγ)(α=β)(γ: -OH)	2015	Acetone
132.96	110.85	151.69	149.15	119.57	120.31	129.87	129.54	63.25	G:4-O-β'	4-O-β'	G-b-GA (αβγ)(α=β)(γ: -OH)	2013t	Acetone
132.84	110.99	151.88	148.55	119.27	120.23	129.87	129.54	63.25	G:4-O-β'	4-O-β'	G-b-GA (αβγ)(α=β)(γ: -OH)	2014e	Acetone
-	113.65	150.86	148.29	116.70	121.57	-	-	-	G:4-O-β'	4-O-β'	G-b-G (G1: C-H))	106	Acetone
-	113.74	151.01	148.43	116.95	121.59	-	-	-	G:4-O-β'	4-O-β'	G-b-G (G1: C-H))	110	Acetone
-	113.40	151.80	149.70	120.06	121.95	-	-	-	G:4-O-β'	4-O-β'	G-b-G (G1: C-H)	102t	Acetone
-	113.50	151.96	149.04	119.70	121.85	-	-	-	G:4-O-β'	4-O-β'	G-b-G (G1: C-H)	101e	Acetone
-	113.40	151.80	149.70	115.21	121.95	-	-	-	G:4-O-β'	4-O-β'	G-b-G (G1: C-H)	102t	Acetone
-	113.27	151.65	149.60	119.66	121.91	-	-	-	G:4-O-β'	4-O-β'	S-b-G (G1: C-H)	90	Acetone
134.04	112.34	150.40	152.27	114.72	123.46	195.94	26.30	-	G:4-O-β'	4-O-β'	S-b-G (αβ)(α: C=O)(β: -CH3)	81	Acetone
131.98	112.10	150.23	152.98	113.40	123.39	193.34	26.30		G:4-O-β'	4-O-β'	G-b-G (αβ)(α: C=O)(β: -CH3)	52	Acetone

Table S6. ^{13}C chemical shifts of G lignin in type-C linkage. Unavailable site is indicated as "-". All values are from the lignin database by Ralph and coworkers(8), referenced on TMS scale. Letters with an apostrophe represent neighboring units.

C1	C2	C3	C4	C5	C6	α	β	γ	Unit Type	Linkage Type	Polymer Note	Compound #	Solvent
135.32	112.24	146.67	143.30	132.91	115.35	37.27	24.52	13.65	β'-5:G:4-O-α'	4-O-α' + 5-β'	G-c-G (αβγ)(α,β:-CH2)(γ: -CH3)	66	DMSO
133.30	110.58	146.71	143.61	131.39	115.23	130.68	122.60	17.25	β'-5:G:4-O-α'	4-O-α' + 5-β'	G-c-G (αβγ)(α=β)(γ: -CH3)	67	DMSO
130.63	110.88	143.59	146.86	128.78	114.82	129.22	128.02	61.52	β'-5:G:4-O-α'	4-O-α' + 5-β'	G-b-S-c-GA (αβγ)(α=β)(γ-OH)	181	DMSO
130.56	110.37	143.72	147.60	129.52	115.37	129.04	128.02	61.70	β'-5:G:4-O-α'	4-O-α' + 5-β'	G-c-GA (αβγ)(α=β)(γ-OH)	2004	DMSO
136.62	113.63	147.52	144.78	134.12	116.39	38.54	25.74	14.08	β'-5:G:4-O-α'	4-O-α' + 5-β'	G-c-G (αβγ)(α,β:-CH2)(γ: -CH3)	66	Acetone
134.43	111.06	147.72	145.03	132.83	115.55	132.05	123.22	18.42	β'-5:G:4-O-α'	4-O-α' + 5-β'	G-c-G (αβγ)(α=β)(γ: -CH3)	67	Acetone
132.20	111.89	145.22	148.82	130.15	116.06	130.40	128.57	63.36	β'-5:G:4-O-α'	4-O-α' + 5-β'	G-b-S-c-GA (αβγ)(α=β)(γ-OH)	181	Acetone
131.91	111.72	145.14	148.84	130.40	116.08	130.54	128.33	63.39	β'-5:G:4-O-α'	4-O-α' + 5-β'	G-c-GA (αβγ)(α=β)(γ-OH)	2004	Acetone
131.95	111.80	145.16	148.95	130.47	116.07	130.52	128.38	63.39	β'-5:G:4-O-α'	4-O-α' + 5-β'	S-c-GA (αβγ)(α=β)(γ-OH)	3063	Acetone
131.24	113.56	145.65	152.41	129.00	119.64	154.10	127.14	193.77	β'-5:G:4-O-α'	4-O-α' + 5-β'	G-c-GA (αβγ)(α=β)(γ: C=O)	2021	Acetone
132.40	113.44	145.81	154.87	131.19	121.43	190.90	-	-	β'-5:G:4-O-α'	4-O-α' + 5-β'	G-c-G (α)(α: C=O)	3061	Acetone

Table S7. ^{13}C chemical shifts of G lignin in type-D linkage. Unavailable site is indicated as “-”. All values are from the lignin database by Ralph and coworkers(8), referenced on TMS scale. Letters and numbers with an apostrophe or double apostrophe represent neighboring units.

C1	C2	C3	C4	C5	C6	α	β	γ	Unit Type	Linkage Type	Polymer Note	Compound #	Solvent
-	109.57	151.68	145.04	131.87	117.93	73.63	32.11	10.49	5'-5:G:4-O-α''	4-O-α'' + 5-5'	DiBenzoDiOxicin (α: -OH)(β:CH2)(γ:Ch3)	277	DMSO
-	109.45	151.68	144.87	132.08	117.93	73.63	32.11	10.49	5'-5:G:4-O-β''	4-O-β'' + 5-5'	DiBenzoDiOxicin (α: -OH)(β:CH2)(γ:Ch3)	277	DMSO
-	108.86	151.72	144.75	132.12	119.35	75.52	33.23	10.71	5'-5:G:4-O-α''	4-O-α'' + 5-5'	DiBenzoDiOxicin (α: -OH)(β:CH2)(γ:Ch3)	277	CDCl3
-	109.47	152.24	145.98	133.19	118.92	75.52	33.23	10.71	5'-5:G:4-O-β''	4-O-β'' + 5-5'	DiBenzoDiOxicin (α: -OH)(β:CH2)(γ:Ch3)	277	CDCl3
-	110.55	153.15	146.73	133.24	119.26	75.52	33.23	10.71	5'-5:G:4-O-α''	4-O-α'' + 5-5'	DiBenzoDiOxicin (α: -OH)(β:CH2)(γ:Ch3)	277	Acetone
-	110.17	152.75	145.95	133.91	119.57	75.52	33.23	10.71	5'-5:G:4-O-β''	4-O-β'' + 5-5'	DiBenzoDiOxicin (α: -OH)(β:CH2)(γ:Ch3)	277	Acetone
134.56	111.88	154.39	152.81	133.72	125.15	191.71	-	-	5'-5:G:4-O-α''	4-O-α'' + 5-5'	DiBenzoDiOxicin (α: C=O)	3062	Acetone
134.42	111.71	154.23	152.46	133.27	125.32	191.66	-	-	5'-5:G:4-O-β''	4-O-β'' + 5-5'	DiBenzoDiOxicin (α: C=O)	3062	Acetone
				C5	C5'								
				131.87	132.08					5-5'	DiBenzoDiOxicin	277	DMSO
				133.19	132.12					5-5'	DiBenzoDiOxicin	277	CDCl3
				133.24	133.91					5-5'	DiBenzoDiOxicin	277	Acetone
				133.72	133.27					5-5'	DiBenzoDiOxicin	3062	Acetone

Table S8. ^{13}C chemical shifts of G lignin in type-E linkage. Unavailable site is indicated as "-". All values are from the lignin database by Ralph and coworkers(8), referenced on TMS scale. Numbers with an apostrophe or double apostrophe represent neighboring units.

C1	C2	C3	C4	C5	C6	α	β	γ	Unit Type	Linkage Type	Polymer Note	Compound #	Solvent
-	113.19	149.79	143.69	118.00	120.25	38.50	24.15	13.68	G:4-O-5'	4-O-5'	G-d-G (αβγ)(α,β: -CH2)(γ: -CH3)	273	DMSO
-	113.43	150.31	142.89	119.39	120.50	37.08	24.18	13.70	G:4-O-5'	4-O-5'	G-d-G (αβγ)(α,β: -CH2)(γ: -CH3)	286	DMSO
-	113.17	149.71	143.77	117.84	120.27	36.96	24.17	13.48	G:4-O-5'	4-O-5'	G-d-G (αβγ)(α,β: -CH2)(γ: -CH3)	287	DMSO
-	111.09	150.45	143.03	119.98	118.81	62.59	-	-	G:4-O-5'	4-O-5'	G-d-G (α)(α-OH)	2039	DMSO
129.43	111.86	149.56	148.05	116.34	121.91	143.63	118.00	167.82	G:4-O-5'	4-O-5'	G-d-G (αβγ)(α=β)(γ: COOH)	3003	DMSO
132.01	111.65	149.09	150.44	115.44	122.51	196.5	26.51	-	G:4-O-5'	4-O-5'	G-d-G (αβ)(α: C=O)(β: -CH3)	300	DMSO
131.84	111.60	149.82	151.24	116.31	124.80	191.57	-	-	G:4-O-5'	4-O-5'	G-d-G (α)(α: C=O)	2038	DMSO
-	114.27	151.45	145.23	119.64	121.35	38.33	25.41	14.05	G:4-O-5'	4-O-5'	G-d-G (αβγ)(α,β: -CH2)(γ: -CH3)	273	Acetone
-	114.37	151.82	144.64	120.54	121.41	38.36	25.39	14.04	G:4-O-5'	4-O-5'	G-d-G (αβγ)(α,β: -CH2)(γ: -CH3)	286	Acetone
-	114.27	151.43	145.27	119.60	121.37	38.34	25.42	14.04	G:4-O-5'	4-O-5'	G-d-G (αβγ)(α,β: -CH2)(γ: -CH3)	287	Acetone
-	112.36	152.34	142.49	122.66	119.76	64.25	-	-	G:4-O-5'	4-O-5'	G-d-G-d-G (α)(α-OH)	2049	Acetone
-	112.52	152.00	144.30	121.17	119.78	64.34	-	-	G:4-O-5'	4-O-5'	G-d-G (α)(α-OH)	2039	Acetone
131.06	112.70	151.38	149.34	118.33	122.80	145.38	117.95	168.02	G:4-O-5'	4-O-5'	G-d-G (αβγ)(α=β)(γ: COOH)	3003	Acetone
131.04	112.66	151.41	149.35	118.36	122.84	144.81	117.93	167.15	G:4-O-5'	4-O-5'	G-d-G (αβγ)(α=β)(γ: COOCH2CH3)	3001	Acetone
133.65	112.68	150.73	151.69	116.80	123.22	196.55	26.47	-	G:4-O-5'	4-O-5'	G-d-G (αβ)(α: C=O)(β: -CH3)	300	Acetone
133.56	112.26	151.49	152.48	117.63	125.55	191.43	-	-	G:4-O-5'	4-O-5'	G-d-G (α)(α: C=O)	2038	Acetone
136.52	104.81	148.46	135.77	144.24	108.57	73.36	32.05	10.06	4'-O-5:G:4-OH	5-O-4'	G-d-G (αβγ)(α-OH)(β: -CH2)(γ: -CH3)	273	DMSO
135.41	103.06	147.59	133.50	146.54	104.51	73.66	32.06	10.06	4'-O-5:G:4-OH	5-O-4'	G-d-G-5,5-G (αβγ)(α-OH)(β: -CH2)(γ: -CH3)	284	DMSO
132.29	107.39	148.71	135.22	144.34	110.95	36.98	24.17	13.67	4'-O-5:G:4-OH	5-O-4'	G-d-G (αβγ)(α,β: -CH2)(γ: -CH3)	287	DMSO
-	107.58	149.28	140.35	142.94	114.05	143.81	116.96	167.82	4'-O-5:G:4-OH	5-O-4'	G-d-G (αβγ)(α=β)(γ: COOH)	3003	DMSO
-	108.09	148.83	143.37	141.97	114.62	195.87	26.32	-	4'-O-5:G:4-OH	5-O-4'	G-d-G (αβ)(α: C=O)(β: -CH3)	300	DMSO
-	108.09	149.47	144.52	142.52	116.27	190.84	-	-	4'-O-5:G:4-OH	5-O-4'	G-d-G (α)(α: C=O)	2038	DMSO
-	107.74	148.93	142.63	145.90	111.54	190.97	-	-	4'-O-5:G:4-OH	5-O-4'	G-d-G (α: C=O)	2039	DMSO
-	105.66	149.29	137.28	145.44	109.81	75.40	33.20	10.48	4'-O-5:G:4-OH	5-O-4'	G-d-G (αβγ)(α-OH)(β: -CH2)(γ: -CH3)	273	Acetone
-	104.50	148.53	135.37	147.29	106.31	75.66	33.11	10.51	4'-O-5:G:4-OH	5-O-4'	G-d-G-5,5-G (αβγ)(α-OH)(β: -CH2)(γ: -CH3)	284	Acetone
133.80	108.23	149.43	136.58	145.58	112.07	38.34	25.42	13.91	4'-O-5:G:4-OH	5-O-4'	G-d-G (αβγ)(α,β: -CH2)(γ: -CH3)	287	Acetone
126.71	108.03	150.12	141.38	144.44	114.46	145.23	117.00	168.05	4'-O-5:G:4-OH	5-O-4'	G-d-G (αβγ)(α=β)(γ: COOH)	3003	Acetone
126.67	107.99	150.12	141.41	144.45	114.45	144.94	116.99	167.16	4'-O-5:G:4-OH	5-O-4'	G-d-G (αβγ)(α=β)(γ: COOCH2CH3)	3001	Acetone
129.73	108.56	149.67	144.09	143.16	115.45	195.93	26.33	-	4'-O-5:G:4-OH	5-O-4'	G-d-G (αβ)(α: C=O)(β: -CH3)	300	Acetone
129.54	108.48	150.37	145.22	143.79	116.99	190.75	-	-	4'-O-5:G:4-OH	5-O-4'	G-d-G (α)(α: C=O)	2038	Acetone
129.00	107.98	149.86	143.92	146.98	112.62	190.91	-	-	4'-O-5:G:4-OH	5-O-4'	G-d-G (α: C=O)	2039	Acetone
128.73	108.40	149.70	143.04	147.16	110.17	191.02	-	-	4'-O-5:G:4-OH	5-O-4'	G-d-G-d-G (α)(α: C=O)	2049	Acetone
134.75	108.03	155.04	138.25	153.71	109.92	191.69	-	-	4'-O-5:G:4-O-5"	4-O-5" + 5-O-4'	G-d-G-d-G (α)(α: C=O)	2049	Acetone

Table S9. ^{13}C chemical shifts of gallic acid and tannic acid. All values are referenced to the TMS scale.

C1	C2	C3	C4	C5	C6	α	β	γ	Compound	Linkage Type	Compound#	Solvent	Reference
121	111	147	141	-	-	166	-	-	Tannic acid	CO-O-C3'	-	-	Zheng et al. 2024(7)
-	110	139	142	146	112	-	-	-	Tannic acid	C3-O-CO'	-	-	Zheng et al. 2024(7)
122	111	145	139	-	-	171	-	-	Gallic acid	-	-	-	Zheng et al. 2024(7)
122.1	110.4	146.5	139.6	-	-	170.5	-	-	Gallic acid	-	-	methanol-d_4	Werner et al. 1997(9)
121.66	110.92	145.35	138.97	145.35	-	170.98	-	-	Gallic acid	-	CID 370	D2O	PubChem CID 370
120.7	109	145.3	137.9	145.3	109	167.8	-	-	Gallic acid	-	bmse000389	D2O	BMRB
120.1	110.1	145.5	139.4	145.5	110.1	168.7	-	-	Gallic acid	-	bmse000389	D2O	BMRB

Supporting References

1. Kennedy JA, Jones GP. Analysis of proanthocyanidin cleavage products following acid-catalysis in the presence of excess phloroglucinol. *J Agric Food Chem.* 2001;49(4):1740-6.
2. Kennedy JA, Hayasaka Y, Vidal S, Waters EJ, Jones GP. Composition of grape skin proanthocyanidins at different stages of berry development. *J Agric Food Chem.* 2001;49(11):5348-55.
3. Xue Y, Yu C, Kang X. Quantitative and structural characterization of native lignin in hardwood and softwood bark via solid-state NMR spectroscopy. *J Agric Food Chem.* 2024;72(32):18056-66.
4. Newman RH, Porter LJ. Solid state 13C-NMR studies on condensed tannins. Plant polyphenols: synthesis, properties, significance: Springer; 1992. p. 339-47.
5. Zhang A, Li J, Zhang S, Mu Y, Zhang W, Li J. Characterization and acid-catalysed depolymerization of condensed tannins derived from larch bark. *Rsc Advances.* 2017;7(56):35135-46.
6. Zeller WE, Ramsay A, Ropiak HM, Fryganas C, Mueller-Harvey I, Brown RH, et al. 1H–13C HSQC NMR spectroscopy for estimating procyanidin/prodelphinidin and cis/trans-flavan-3-ol ratios of condensed tannin samples: Correlation with thiolysis. *J Agric Food Chem.* 2015;63(7):1967-73.
7. Zheng Z, Schmidt-Rohr K. Phenolic syringyl end groups in 13C-enriched hardwoods detected and quantified by solid-state NMR. *Solid State Nucl Magn Reson.* 2024;133:101947.
8. Ralph SA, Ralph J, Landucci L, Landucci L. NMR database of lignin and cell wall model compounds. US Forest Prod Lab, Madison, WI (http://ars usda gov/Services/docs htm. 2004.
9. Werner I, Bacher A, Eisenreich W. Retrobiosynthetic NMR studies with 13C-labeled glucose: formation of gallic acid in plants and fungi. *J Biol Chem.* 1997;272(41):25474-82.
